# Supplementary material for: Synthesis and in vitro anticancer activities of substituted N-(4′-nitrophenyl)-l-prolinamides
Source: R Soc Open Sci. 2020 Sep 9;7(9):200906. doi: 10.1098/rsos.200906 (PMC7540745; doi:10.1098/rsos.200906)

Synthesis and *in vitro* Anticancer Activities of Substituted *N*-(4'-Nitrophenyl)-L-prolinamides

Adejoke Osinubi,[a,b,c] Josephat Izunobi,[a] Xiaoguang Bao,[b]* Olayinka Asekun,[a] Jiehong Kong,[d] Chunshan Gui[d] and Oluwole Familoni[a]*

[a]Department of Chemistry, University of Lagos, Akoka-Yaba, Lagos, Nigeria
[b]College of Chemistry, Chemical Engineering and Material Science, Soochow University Suzhou, Jiangsu 215123, China
[c]Chemical Sciences Department, College of Science & Information Technology, Tai Solarin University of Education, P.M.B. 2118, Ijebu Ode, Ogun State, Nigeria
[d]College of Pharmaceutical Sciences, Soochow University, Suzhou 215123, P. R. China

*Email: familonio@unilag.edu.ng and xgbao@suda.edu.cn

SUPPORTING INFORMATION

Table of Content

^{1}H- & ^{13}C-NMR spectra of *N*-(4'-Nitrophenyl)-L-prolines

- *N*-(4'-Nitrophenyl)-L-proline (**3a**) …… S2
- *N*-(4'-Cyanophenyl)-L-proline (**3b**) …… S3
- 4-Hydroxy-*N*-(4'-nitrophenyl)-L-proline (**3c**) …… S4

^{1}H- & ^{13}C-NMR spectra of *N*-(4'-Nitrophenyl)-L-prolinamides

- *N',N'*-Dibutyl-*N*-(4'-nitrophenyl)-L-prolinamide (**4a**) …… S5
- *N'*-Butyl-*N*-(4'-nitrophenyl)-L-prolinamide (**4b**) …… S6
- *N*-(4'-Nitrophenyl)-*N'*-propyl-L-prolinamide (**4c**) …… S7
- *N*-(4'-Nitrophenyl)-*N'*-(1''-prop-1''-ynyl)-L-prolinamide (**4d**) …… S8
- *N'*-Cyclohexyl-*N*-(4'-nitrophenyl)-L-prolinamide (**4e**) …… S9
- *N*-(4'-Nitrophenyl)-*N'*-(phenylsulphonyl)-L-prolinamide (**4f**) …… S10
- *N'*-(*tert*-Butyl)-*N*-(4'-nitrophenyl)-L-prolinamide (**4g**) …… S11
- *N*-(4'-Nitrophenyl)-*N'*-(4''-tosyl)-L-prolinamide (**4h**) …… S12
- 4''-Morpholinyl *N*-(4'-nitrophenyl)-2-pyrrolidinyl ketone (**4i**) …… S13
- *N',N'*-Diisopropyl-*N*-(4'-nitrophenyl)-L-prolinamide (**4j**) …… S14
- *N*-(4'-Nitrophenyl)-*N'*-(4''-tolyl)-L-prolinamide (**4k**) …… S15
- *N*-(4'-Cyanophenyl)-*N'*-(4''-tolyl)-L-prolinamide (**4l**) …… S16
- *trans*-4-Hydroxy-*N*-(4'-nitrophenyl)-*N'*-(4''-tolyl)-L-prolinamide (**4m**) …… S17
- 2''-Isoindolinyl-*N*-(4'-nitrophenyl)-2-pyrrolidinyl ketone (**4n**) …… S18
- *N*-(4'-Nitrophenyl)-*N',N'*-dipropyl-L-prolinamide (**4o**) …… S19
- *N*-(4'-Nitrophenyl)-*N'*-(2''-pyridinyl)-L-prolinamide (**4p**) …… S20
- *N*'-Benzyl-*N*-(4'-nitrophenyl)-L-prolinamide (**4q**) …… S21
- *N'*-(2''-Cyanophenyl)-*N*-(4'-nitrophenyl)-L-prolinamide (**4r**) …… S22
- *N'*-Methyl-*N*-(4'-nitrophenyl)-*N'*-phenyl-L-prolinamide (**4s**) …… S23
- *N'*-(2''-(Hydroxymethyl)phenyl)-*N*-(4'-nitrophenyl)-L-prolinamide (**4t**) …… S24
- *N,N'*-*bis*(4'-Nitrophenyl)-L-prolinamide (**4u**) …… S25
- *N'*-Isopropyl-*N*-(4'-nitrophenyl)-L-prolinamide (**4v**) …… S26
- *N',N'*-Dicyclohexyl-*N*-(4'-nitrophenyl)-L-prolinamide (**4w**) …… S27

^{1}H-NMR spectrum of *N*-(4’-Nitrophenyl)-L-proline (3a)

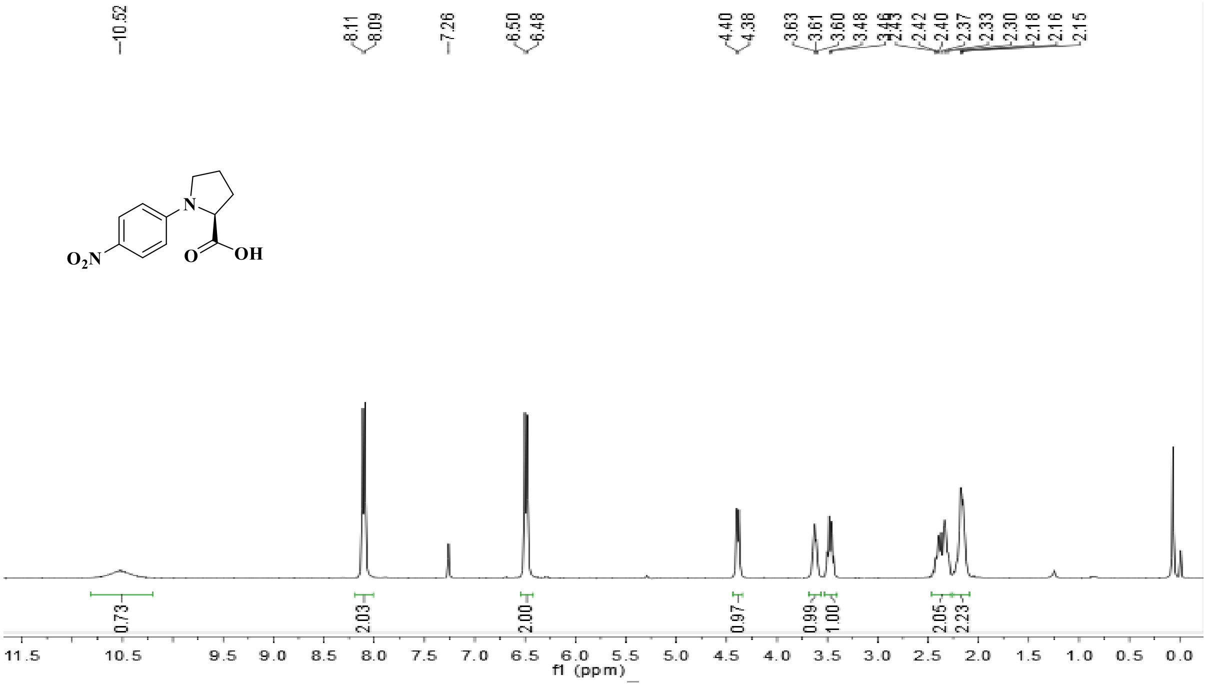

^{13}C-NMR spectrum of *N*-(4’-Nitrophenyl)-L-proline (3a)

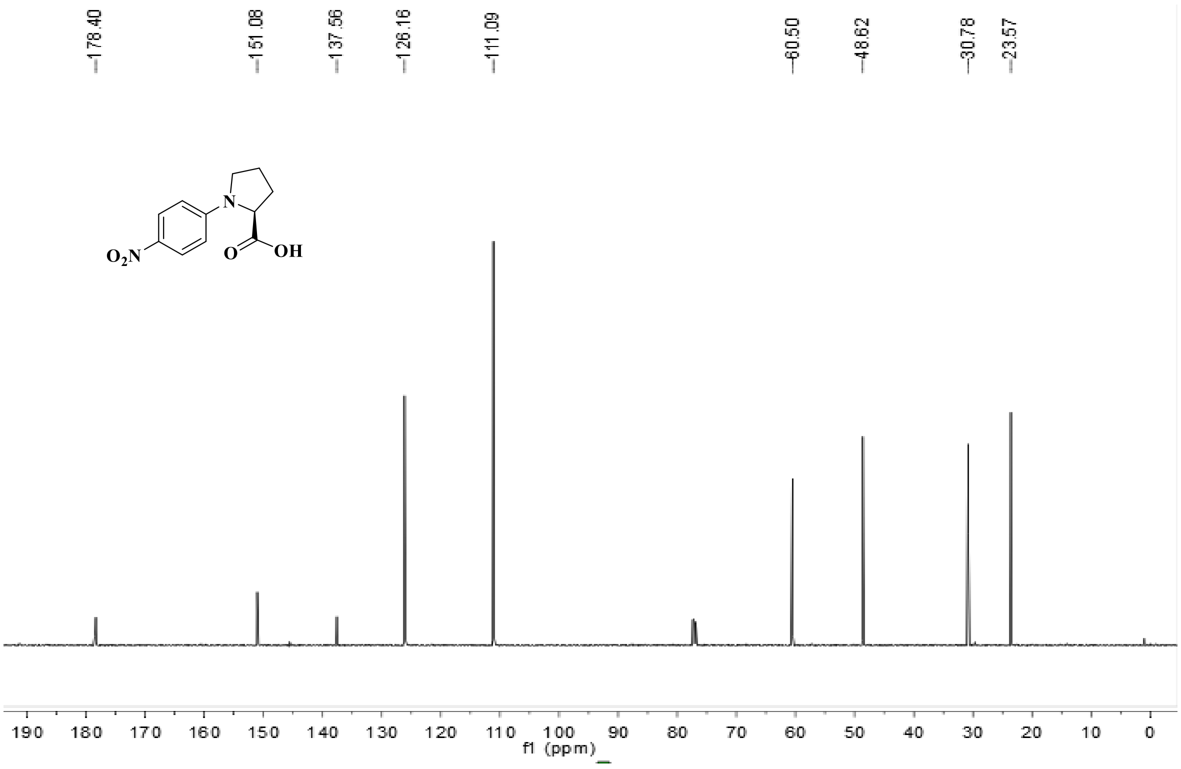

^{1}H-NMR spectrum of *N*-(4’-Cyanophenyl)-L-proline (3b)

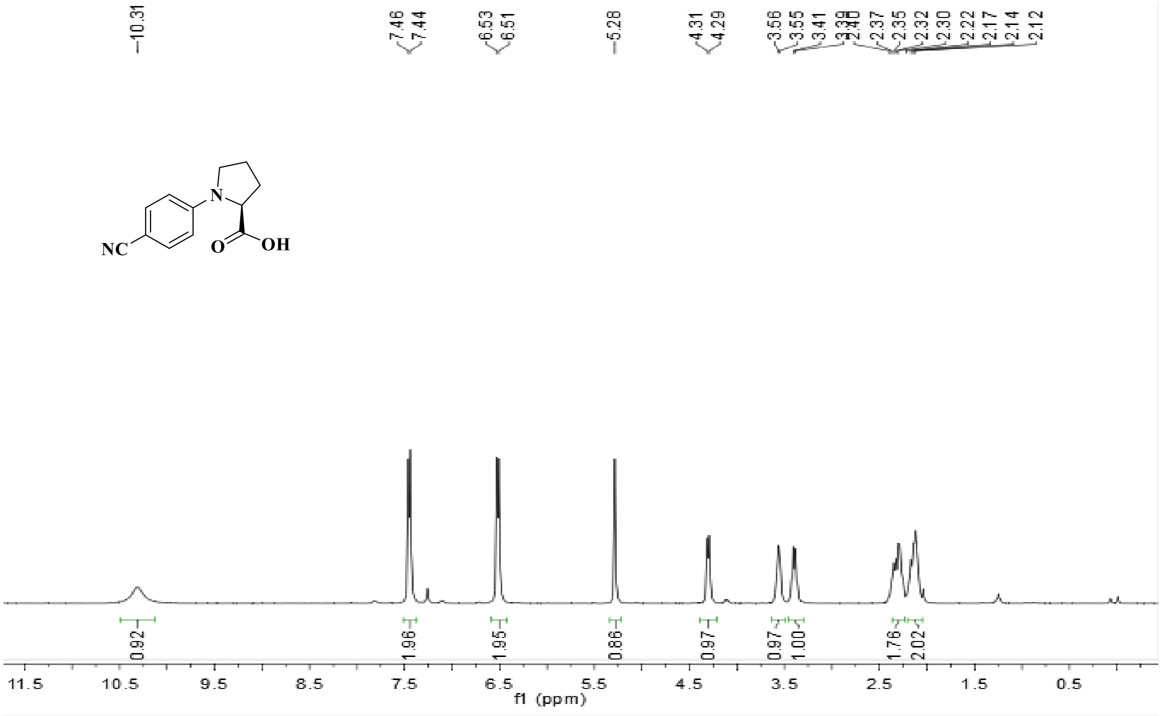

^{13}C-NMR spectrum of *N*-(4’-Cyanophenyl)-L-proline (3b)

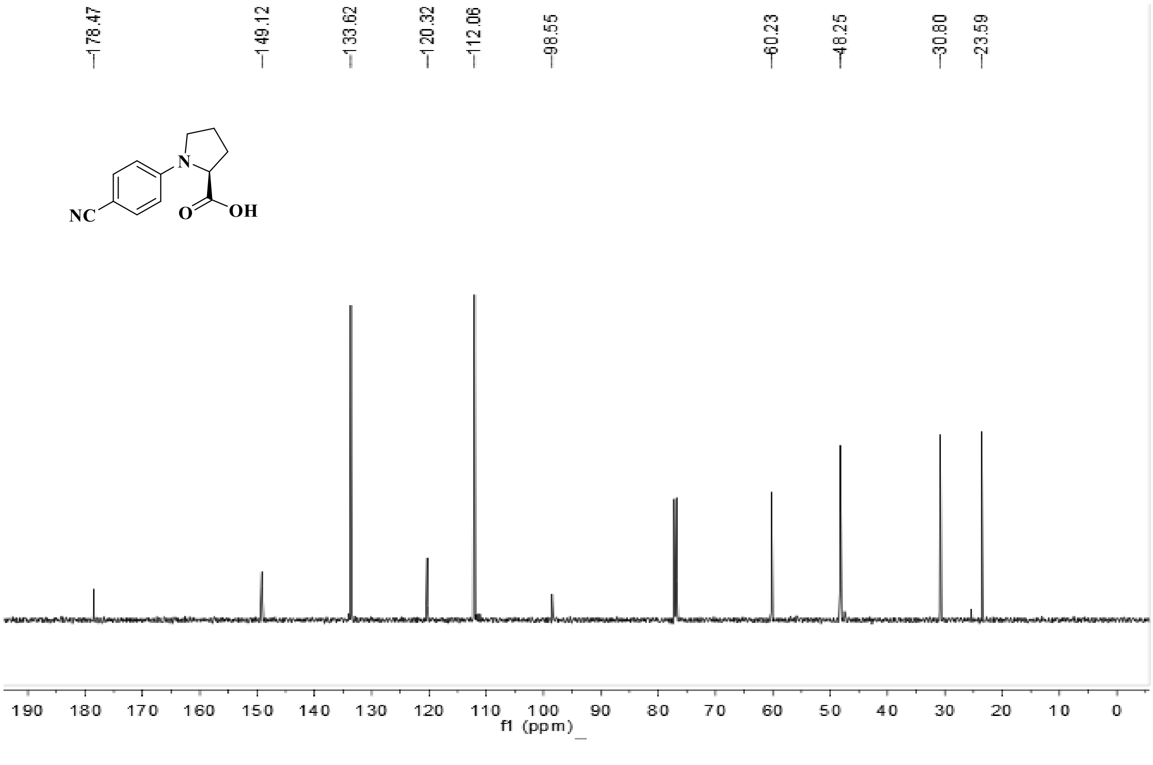

^{1}H-NMR spectrum of 4-Hydroxy-*N*-(4’-nitrophenyl)-L-proline (3c)

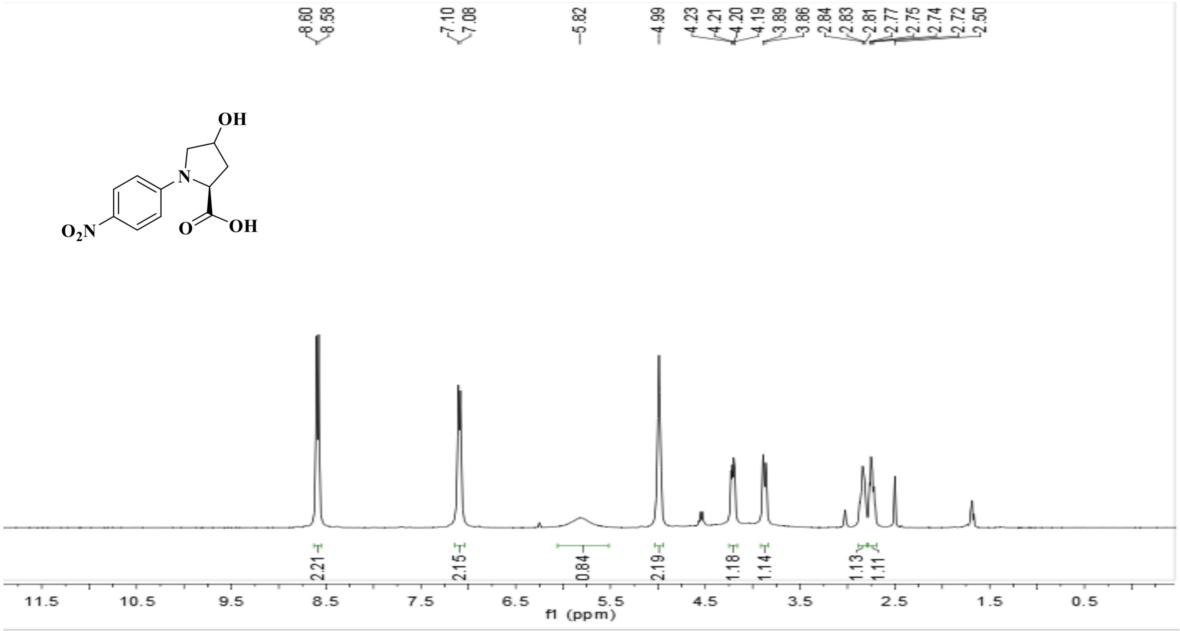

^{13}C-NMR spectrum of 4-Hydroxy-*N*-(4’-nitrophenyl)-L-proline (3c)

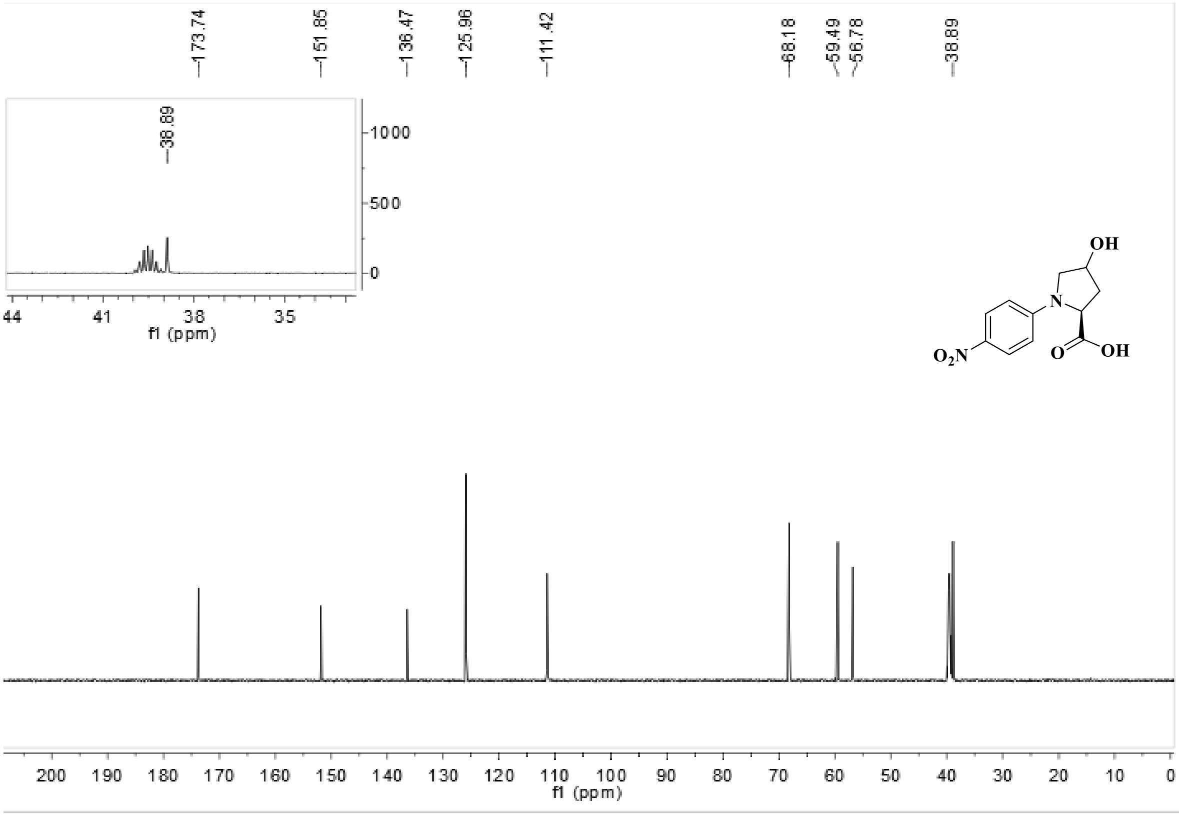

^{1}H-NMR spectrum of *N'*,*N'*-Dibutyl-*N*-(4'-nitrophenyl)-L-prolinamide (4a)

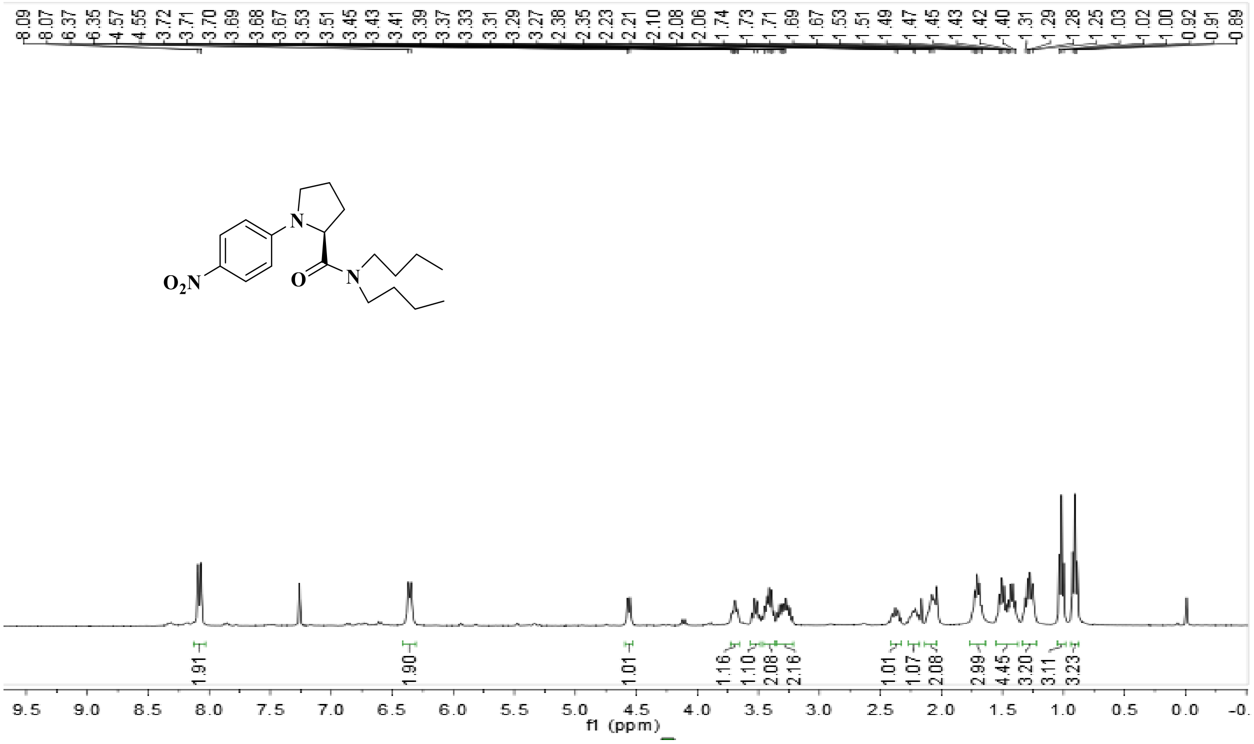

^{13}C-NMR spectrum of *N'*,*N'*-Dibutyl-*N*-(4'-nitrophenyl)-L-prolinamide (4a)

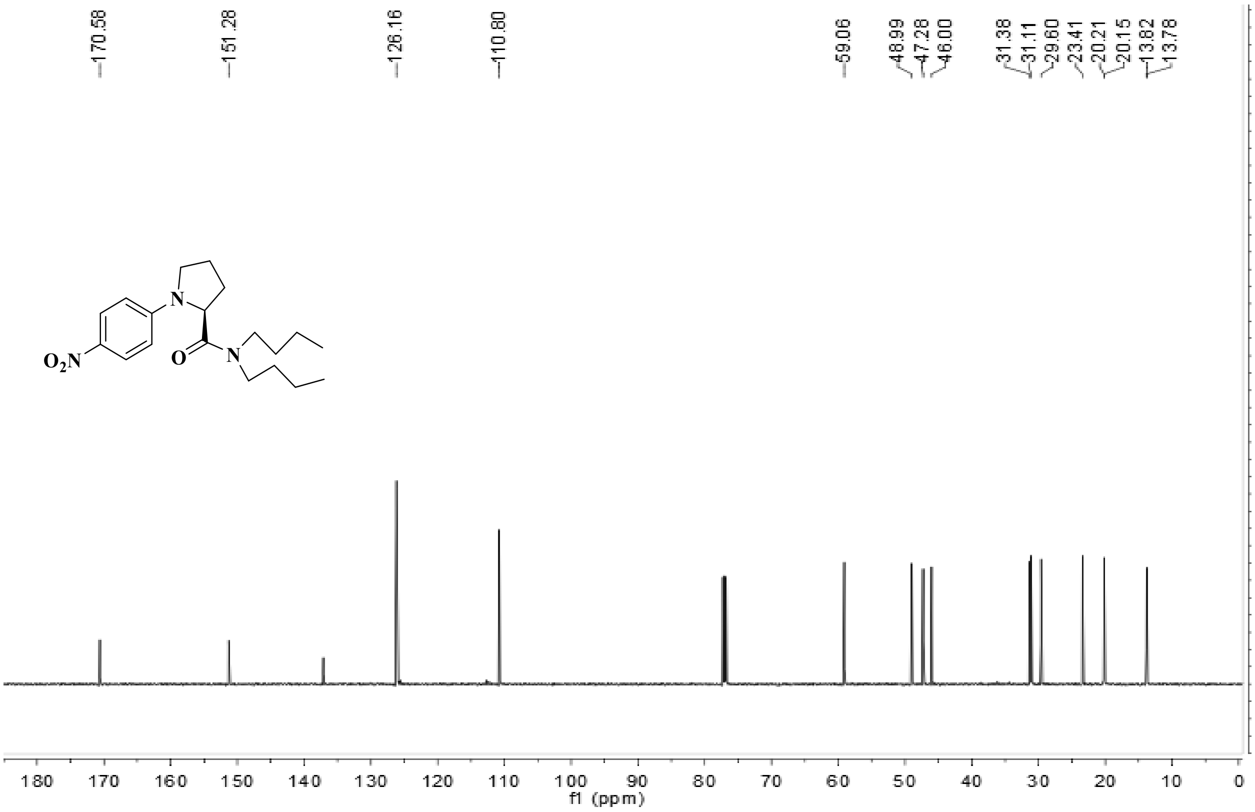

^{1}H-NMR spectrum of *N'*-Butyl-*N*-(4'-nitrophenyl)-L-prolinamide (4b)

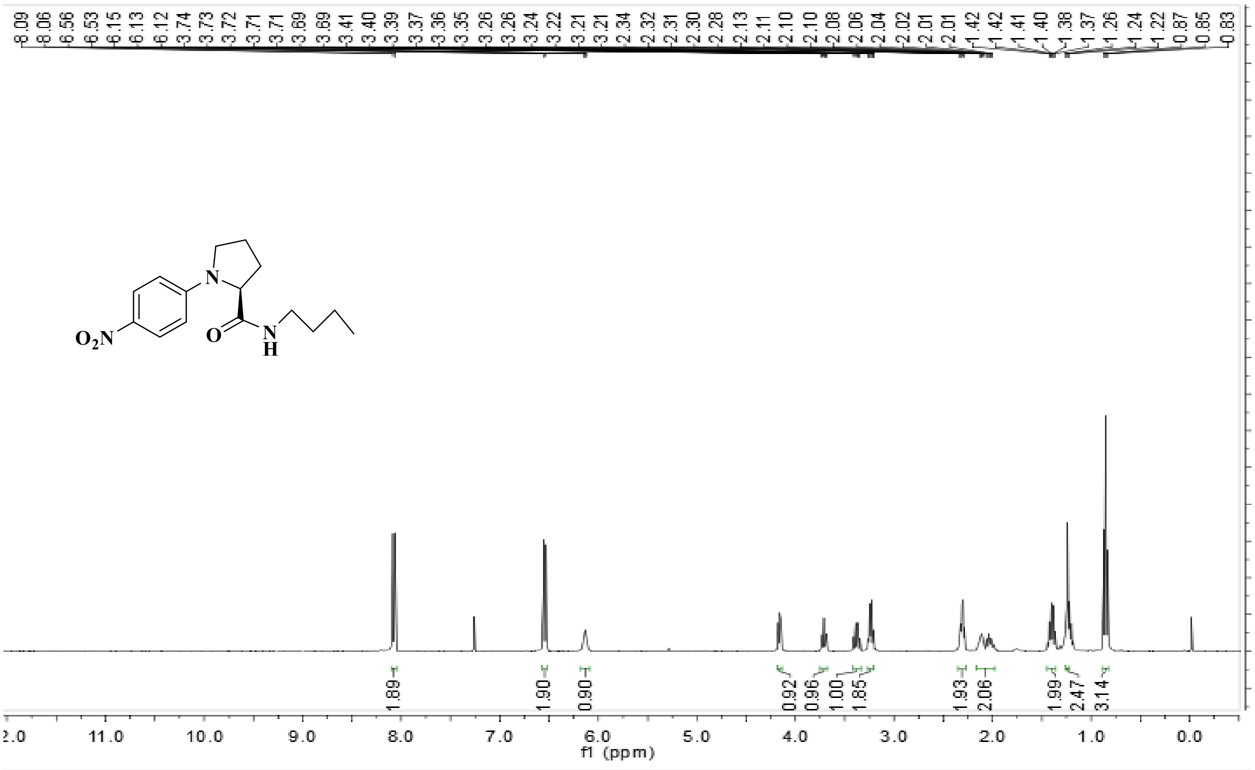

^{13}C-NMR spectrum of *N'*-Butyl-*N*-(4'-nitrophenyl)-L-prolinamide (4b)

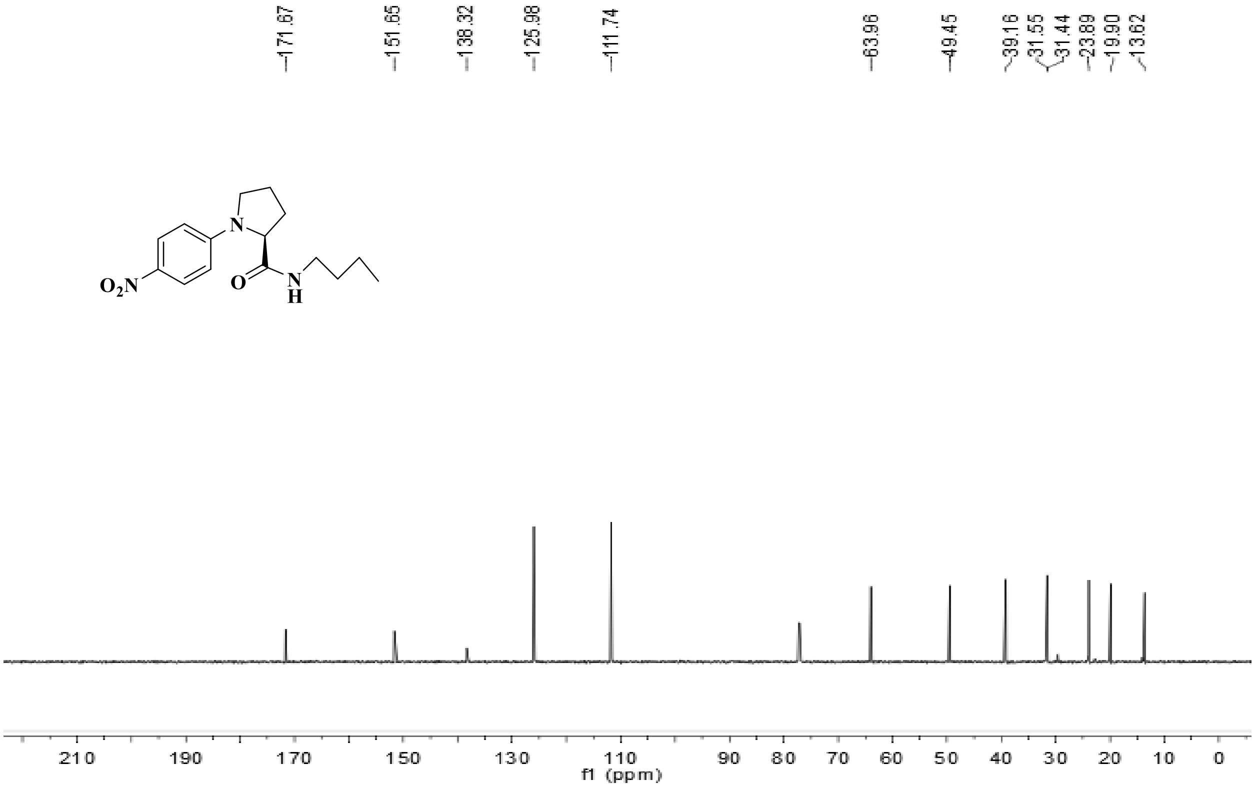

^{1}H-NMR spectrum of *N*-(4'-Nitrophenyl)-*N'*-propyl-L-prolinamide (4c)

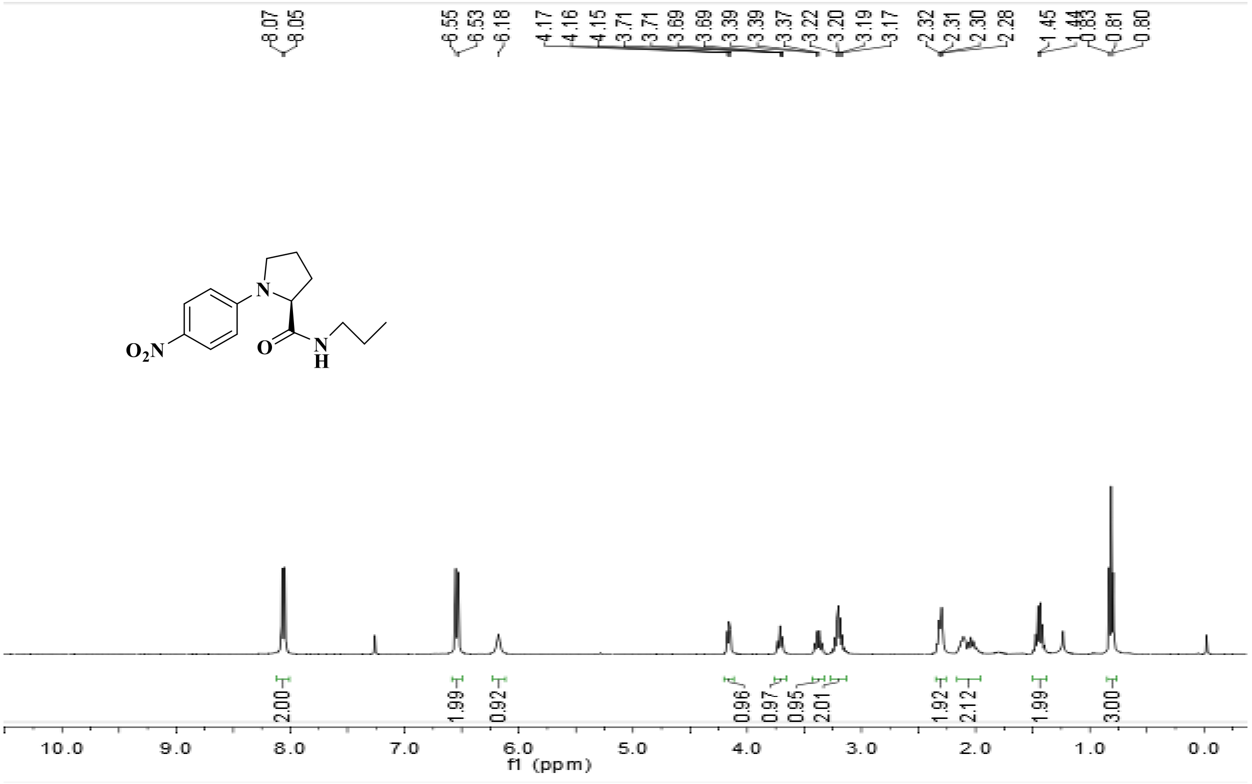

^{13}C-NMR spectrum of *N*-(4'-Nitrophenyl)-*N'*-propyl-L-prolinamide (4c)

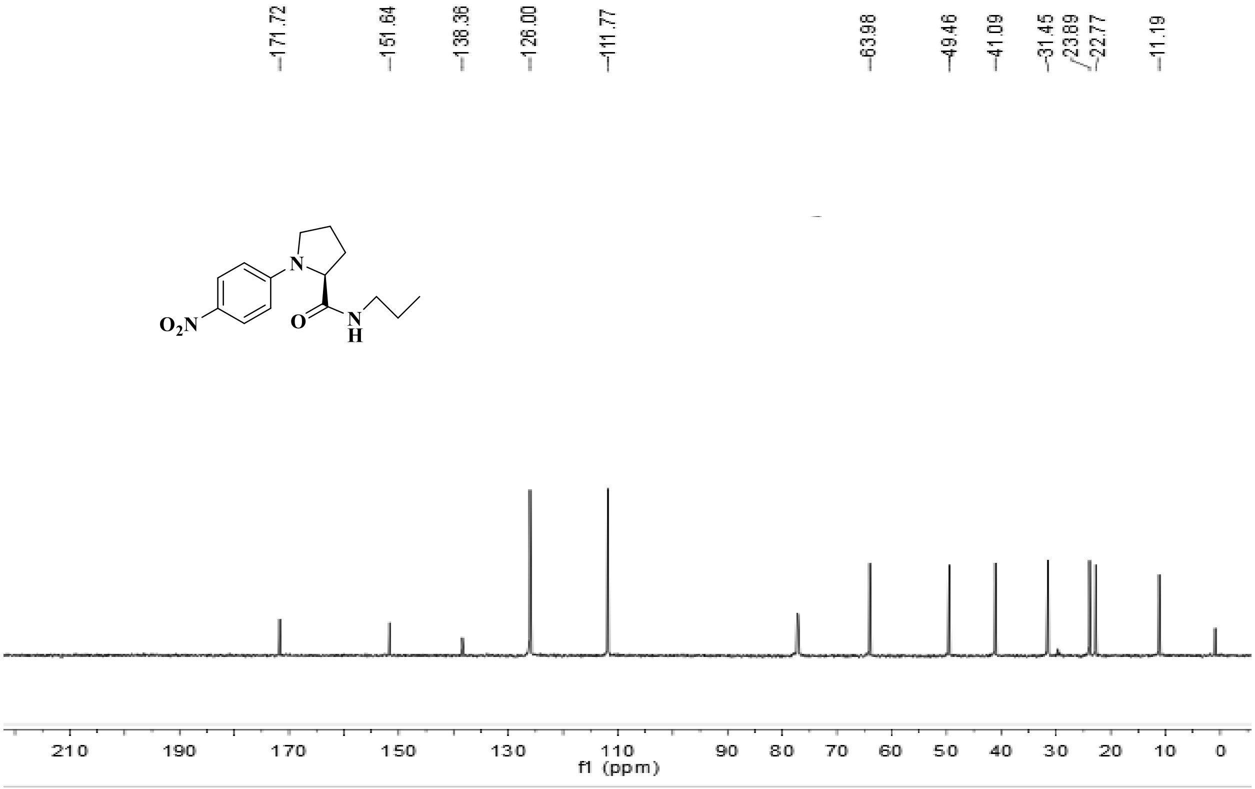

^{1}H-NMR spectrum of *N*-(4’-Nitrophenyl)-*N’*-(1’’-prop-1’’-ynyl)-L-prolinamide (4d)

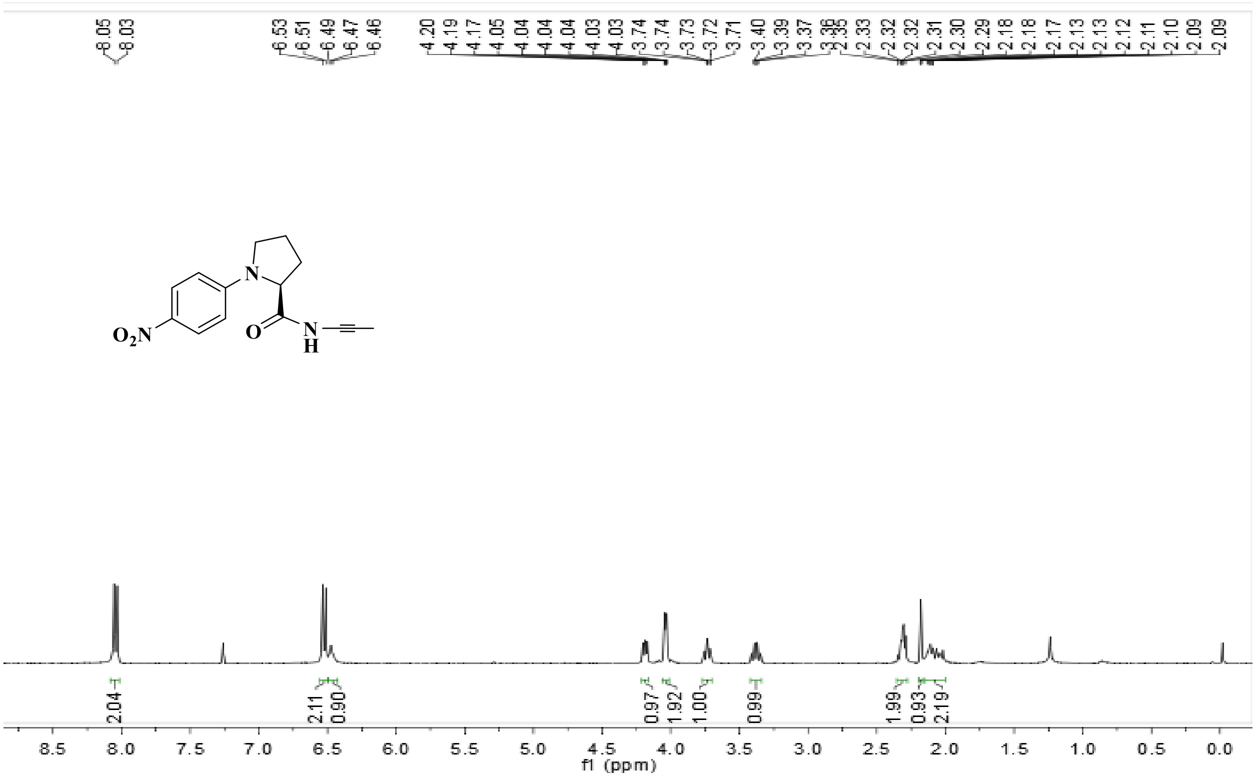

^{13}C-NMR spectrum of *N*-(4’-Nitrophenyl)-*N’*-(1’’-prop-1’’-ynyl)-L-prolinamide (4d)

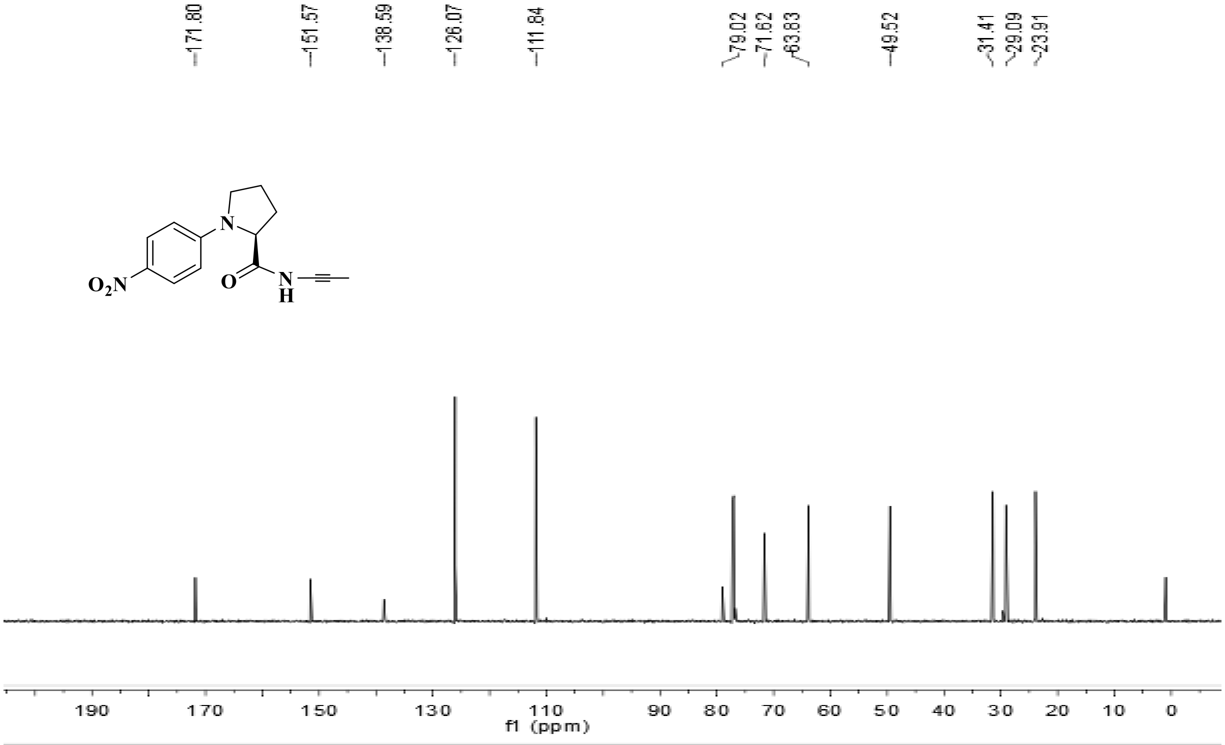

^{1}H-NMR spectrum of *N'*-Cyclohexyl-*N*-(4'-nitrophenyl)-L-prolinamide (4e)

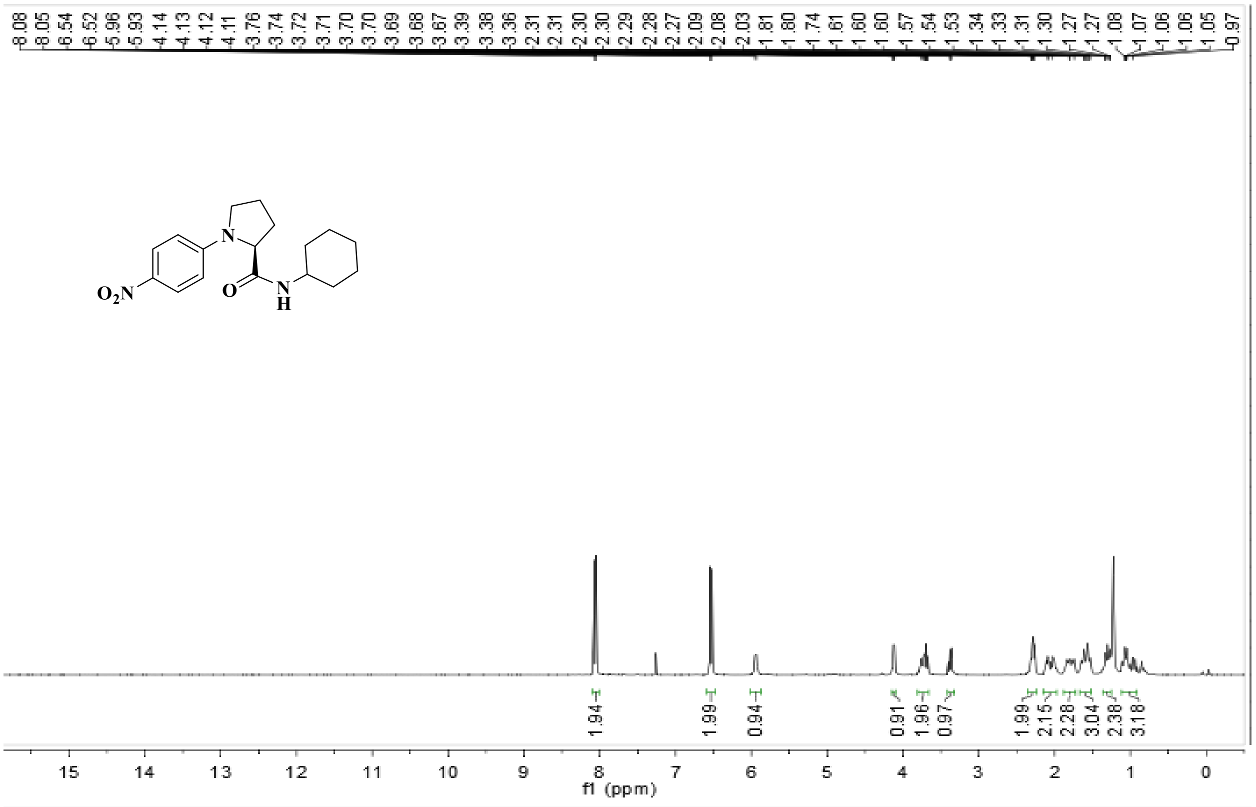

^{13}C-NMR spectrum of *N'*-Cyclohexyl-*N*-(4'-nitrophenyl)-L-prolinamide (4e)

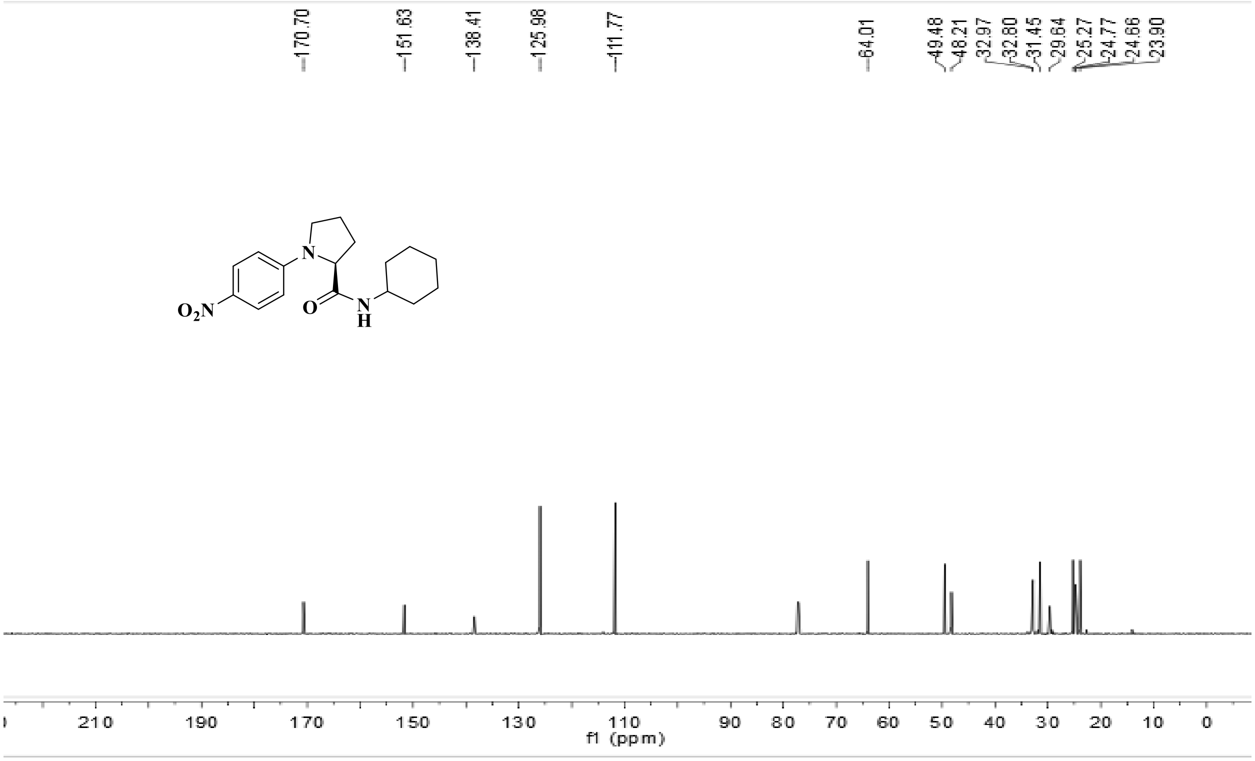

^{1}H-NMR spectrum of *N*-(4’-Nitrophenyl)-*N’*-(phenylsulphonyl)-L-prolinamide (4f)

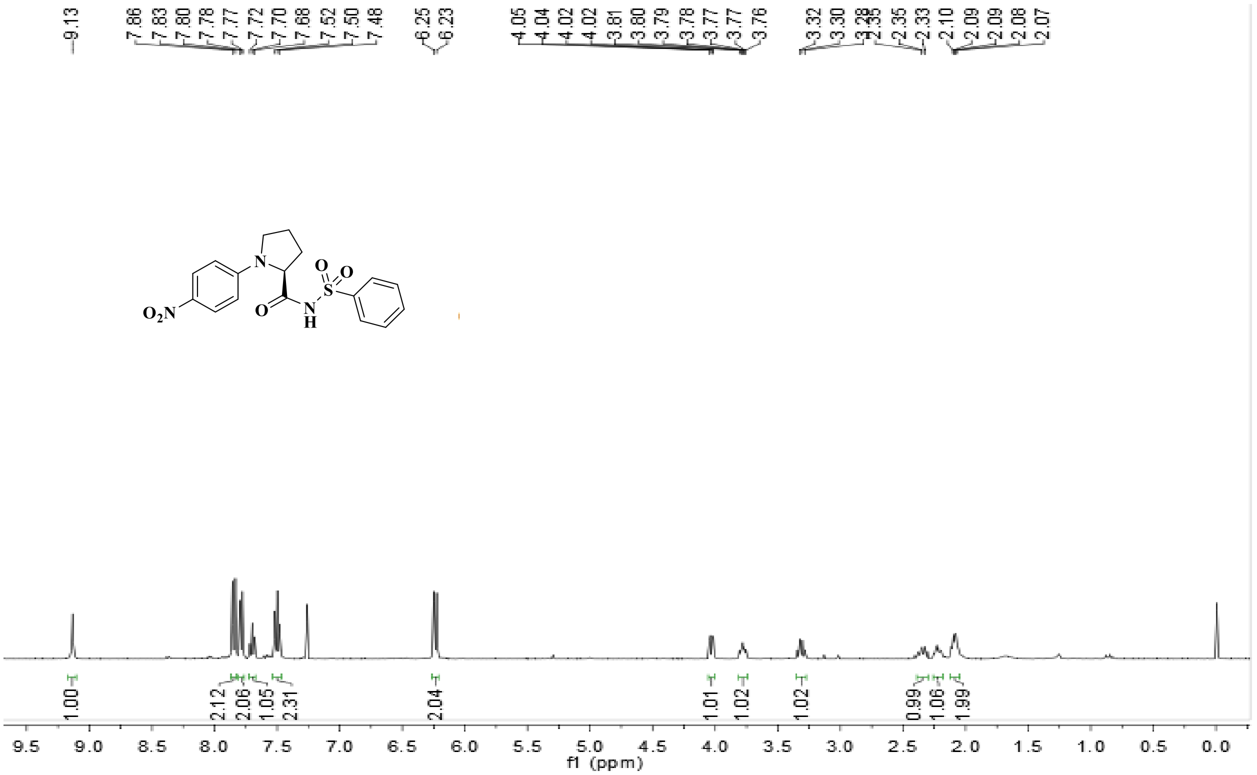

^{13}C-NMR spectrum of *N*-(4’-Nitrophenyl)-*N’*-(phenylsulphonyl)-L-prolinamide (4f)

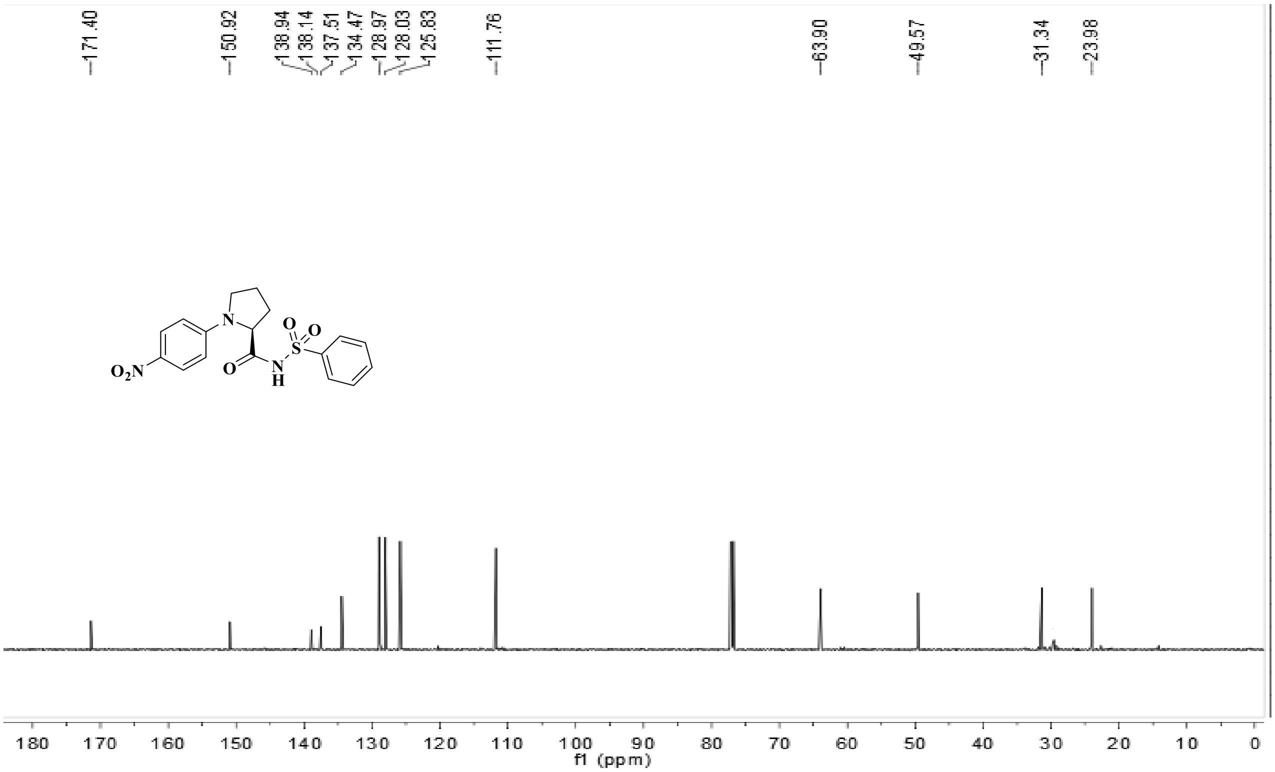

^{1}H-NMR spectrum of *N'*-(*tert*-Butyl)-*N*-(4'-nitrophenyl)-L-prolinamide (4g)

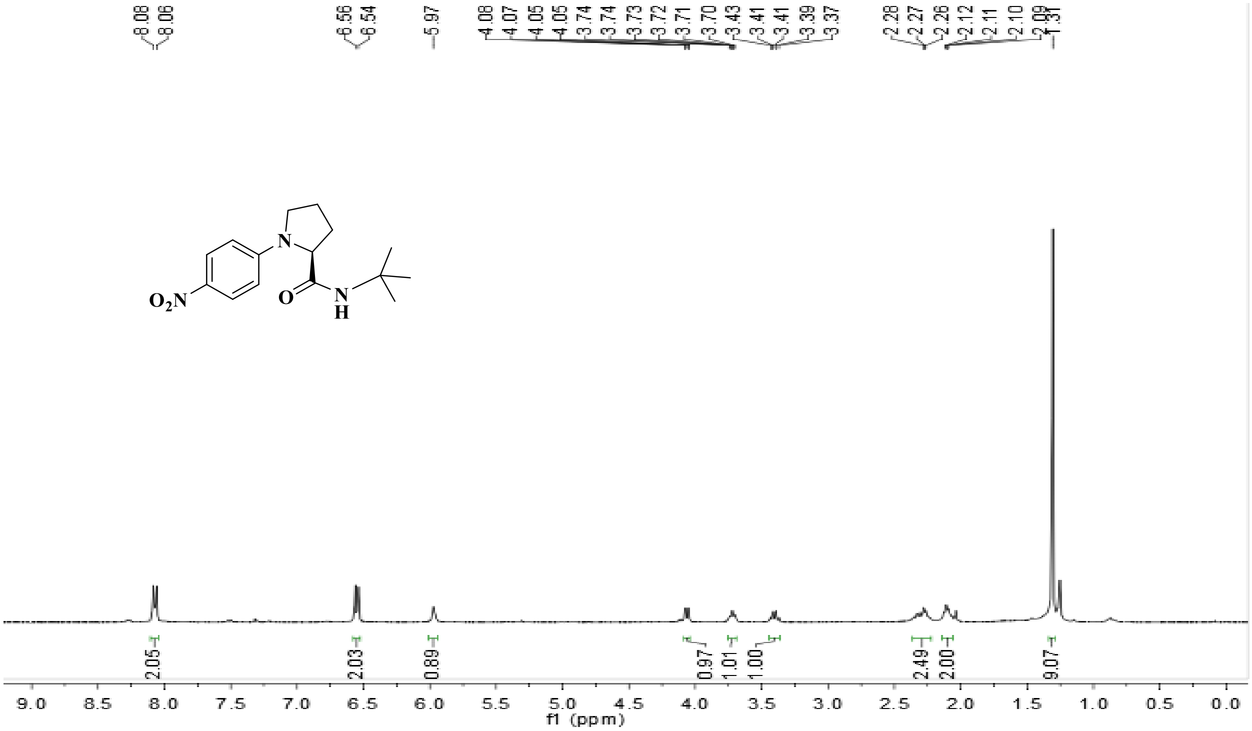

^{13}C-NMR spectrum of *N'*-(*tert*-Butyl)-*N*-(4'-nitrophenyl)-L-prolinamide (4g)

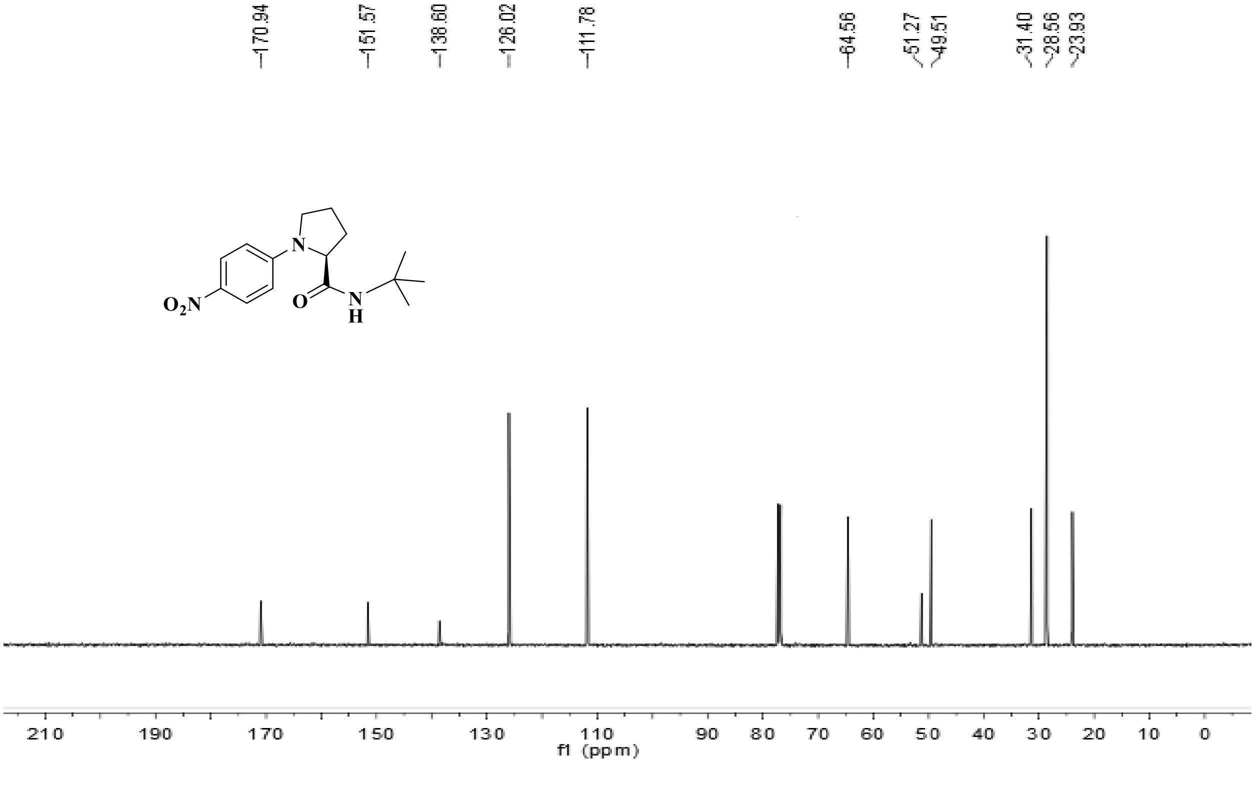

^{1}H-NMR spectrum of *N*-(4’-Nitrophenyl)-*N’*-(4’’-tosyl)-L-prolinamide (4h)

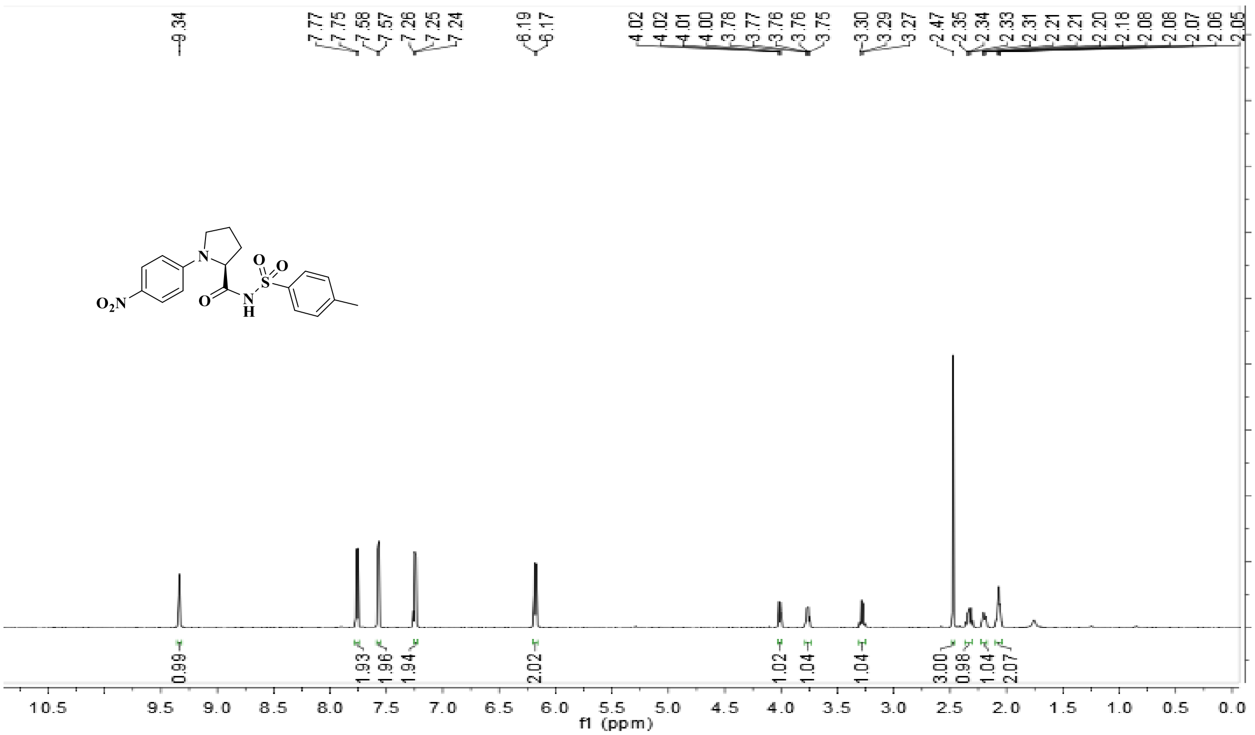

^{13}C-NMR spectrum of *N*-(4’-Nitrophenyl)-*N’*-(4’’-tosyl)-L-prolinamide (4h)

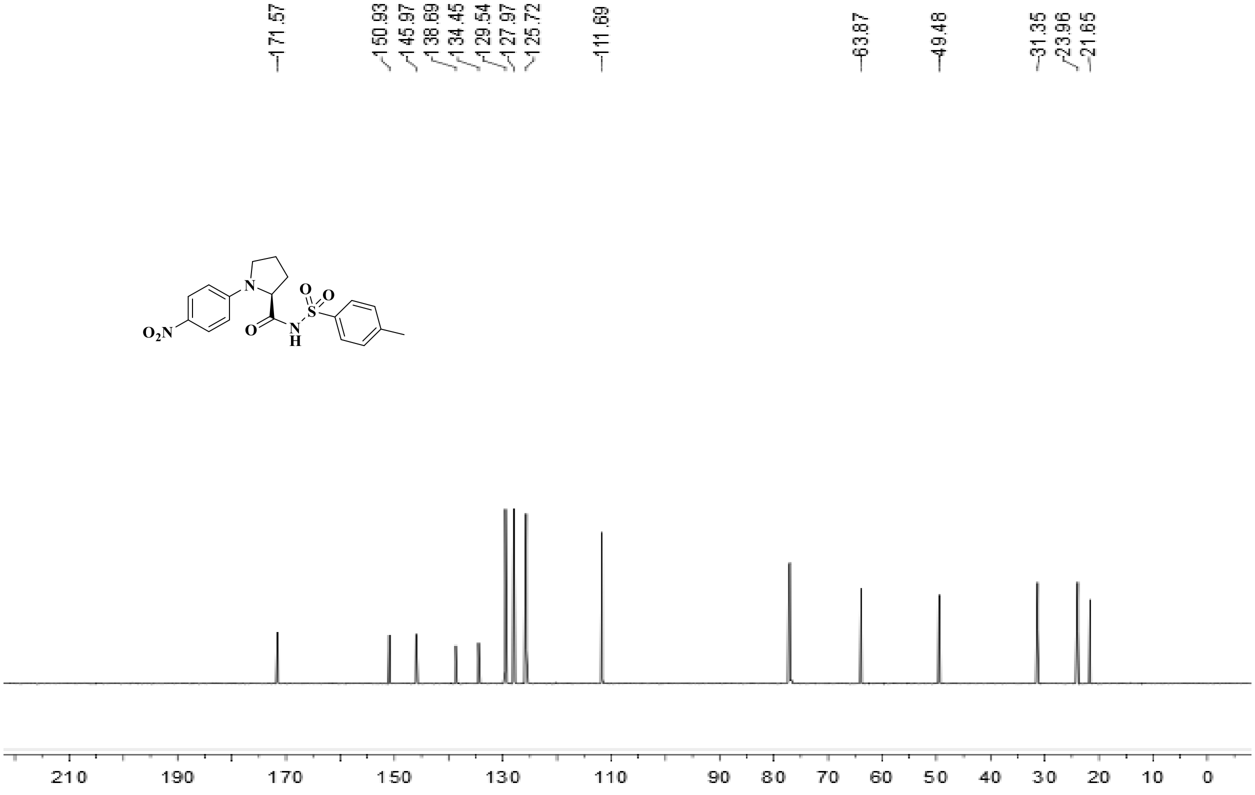

^{1}H-NMR spectrum of 4’’-Morpholinyl *N*-(4’-nitrophenyl)-2-pyrrolidinyl ketone (4i)

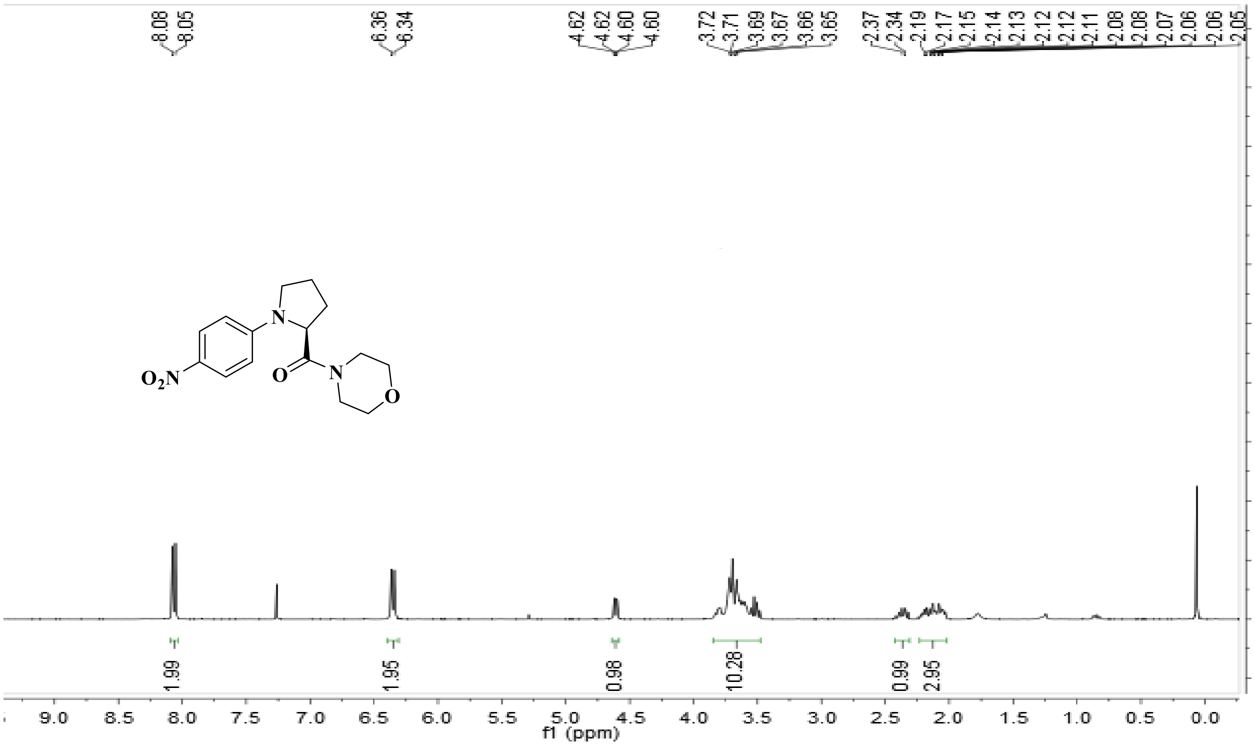

^{13}C-NMR spectrum of 4’’-Morpholinyl *N*-(4’-nitrophenyl)-2-pyrrolidinyl ketone (4i)

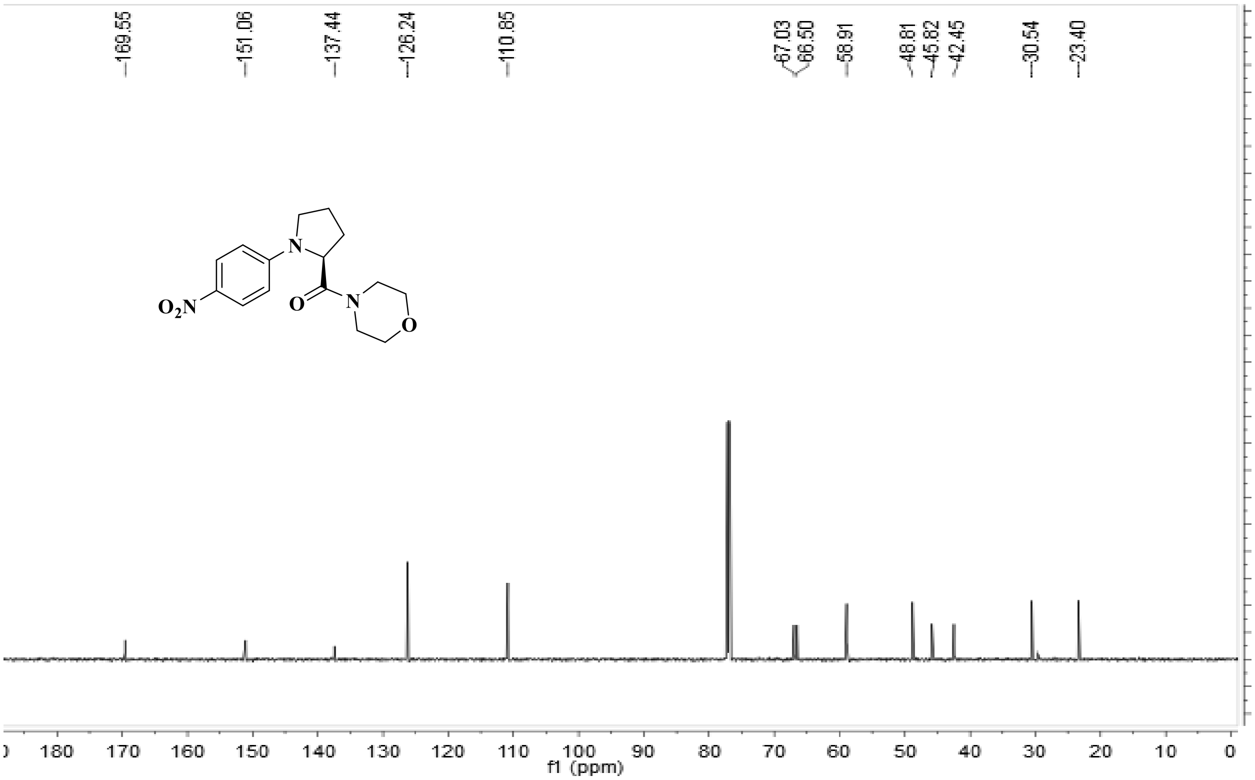

^{1}H-NMR spectrum of *N’*,*N’*-Diisopropyl-*N*-(4’-nitrophenyl)-L-prolinamide (4j)

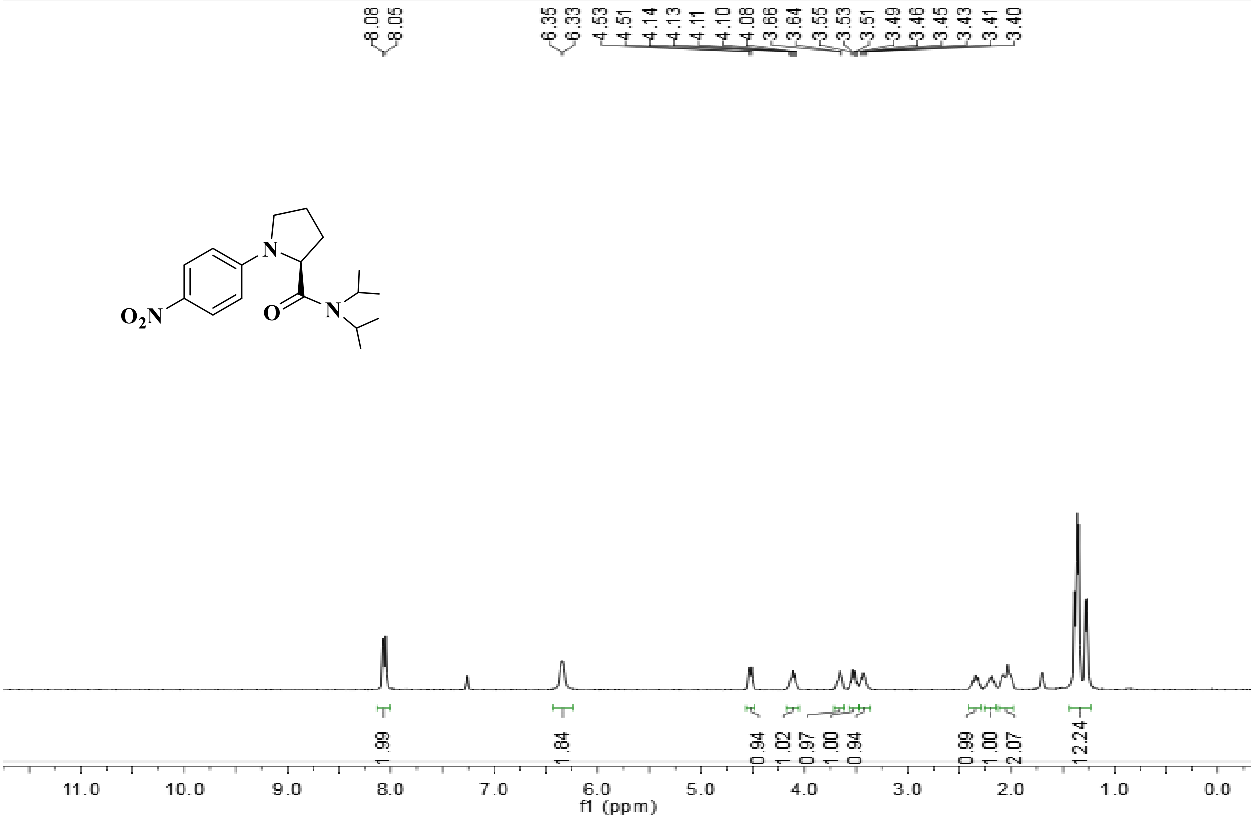

^{13}C-NMR spectrum of *N’*,*N’*-Diisopropyl-*N*-(4’-nitrophenyl)-L-prolinamide (4j)

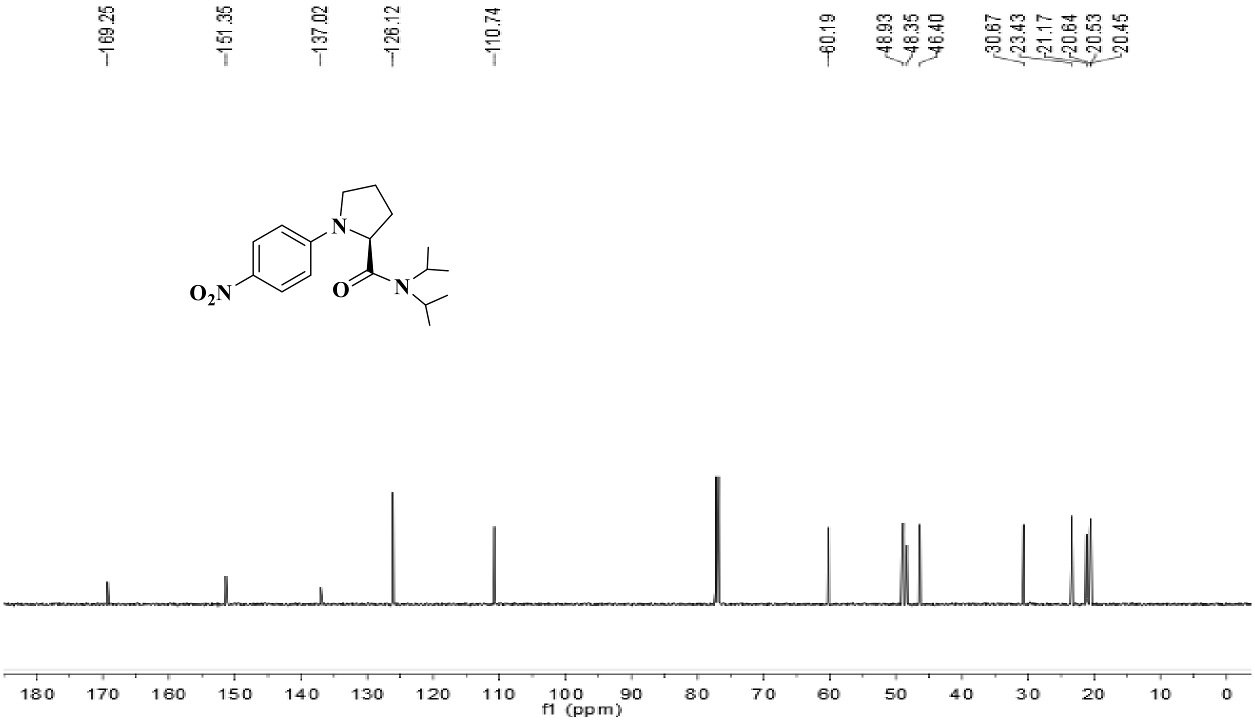

^{1}H-NMR spectrum of *N*-(4’-Nitrophenyl)-*N’*-(4’’-tolyl)-L-prolinamide (4k)

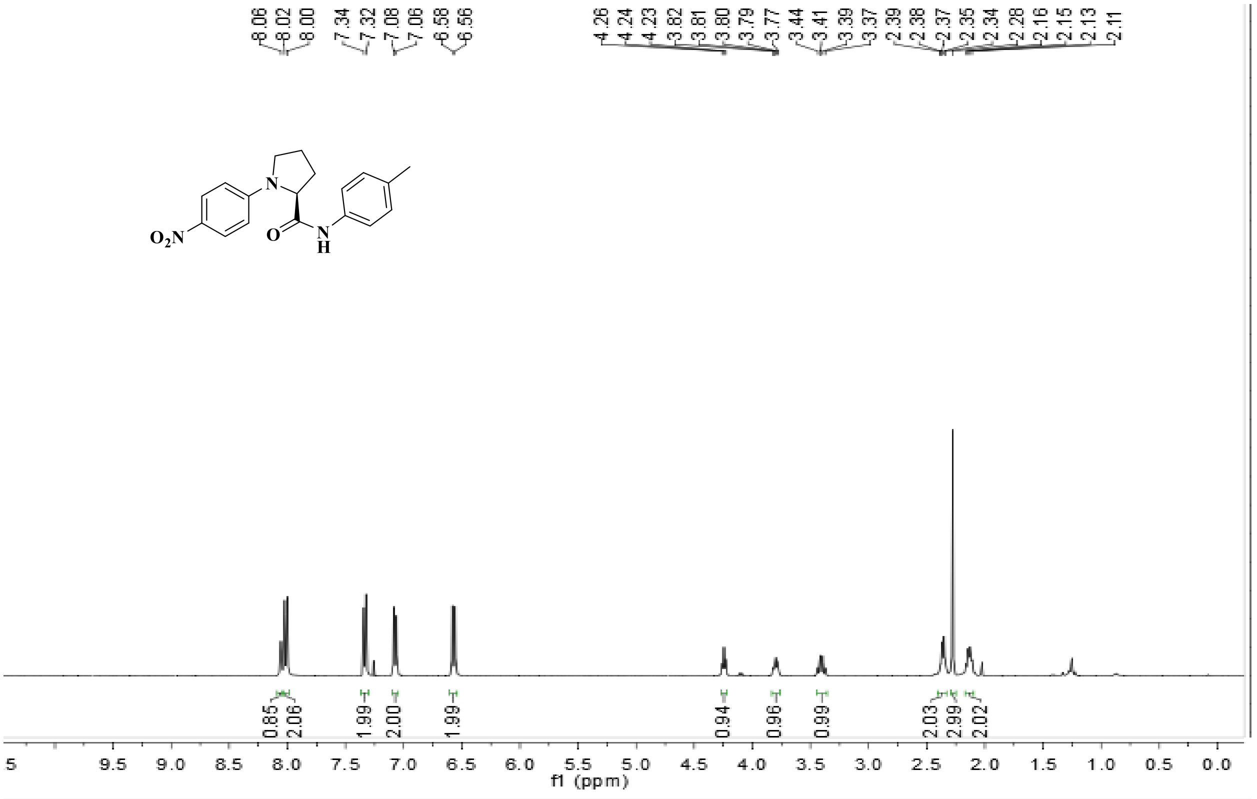

^{13}C-NMR spectrum of *N*-(4’-Nitrophenyl)-*N’*-(4’’-tolyl)-L-prolinamide (4k)

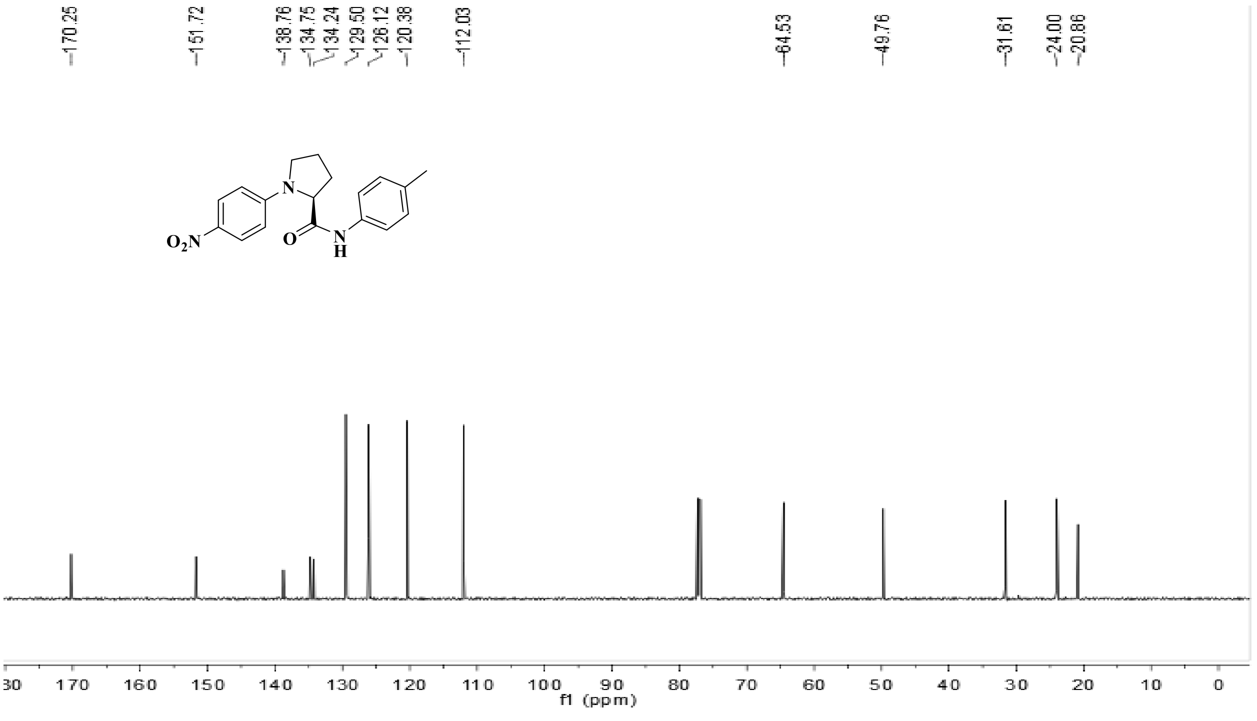

^{1}H-NMR spectrum of *N*-(4'-Cyanophenyl)-*N'*-(4''-tolyl)-L-prolinamide (4l)

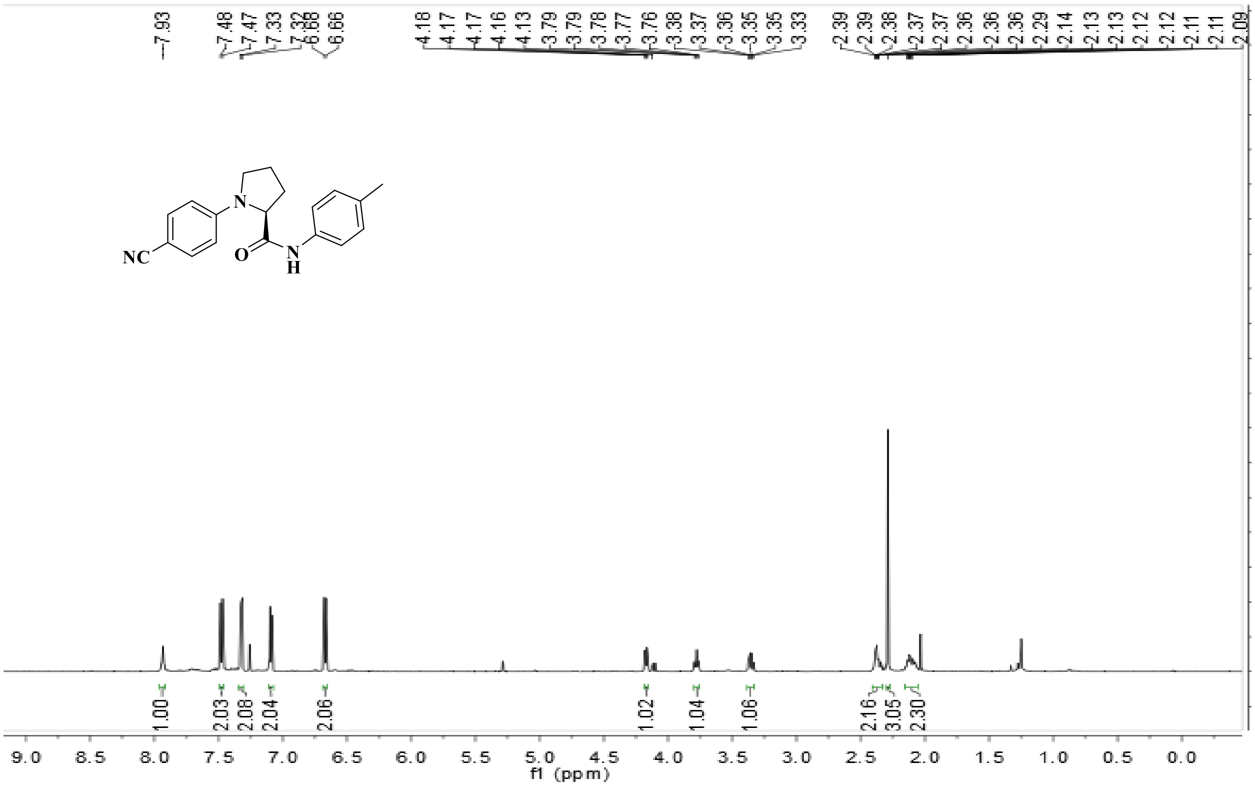

^{13}C-NMR spectrum of *N*-(4'-Cyanophenyl)-*N'*-(4''-tolyl)-L-prolinamide (4l)

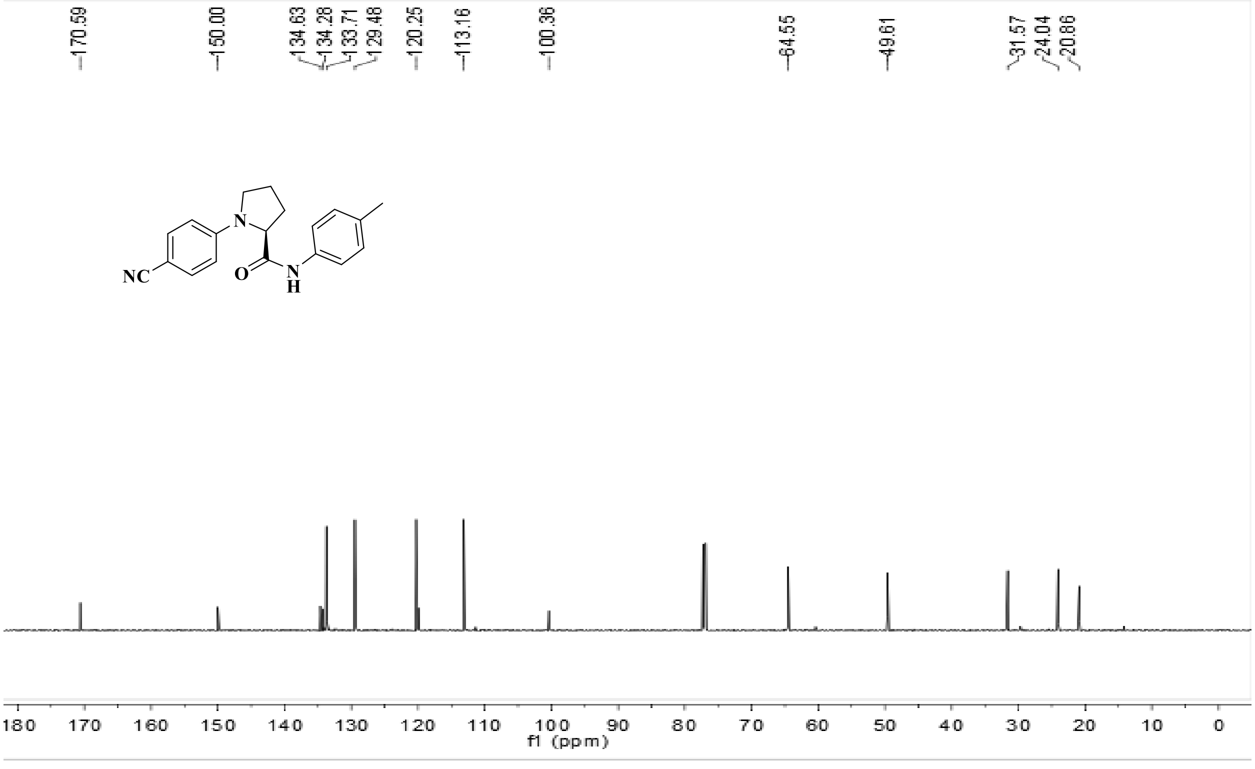

^{1}H-NMR spectrum of *trans*-4-Hydroxy-*N*-(4'-nitrophenyl)-*N'*-(4''-tolyl)-L-prolinamide (4m)

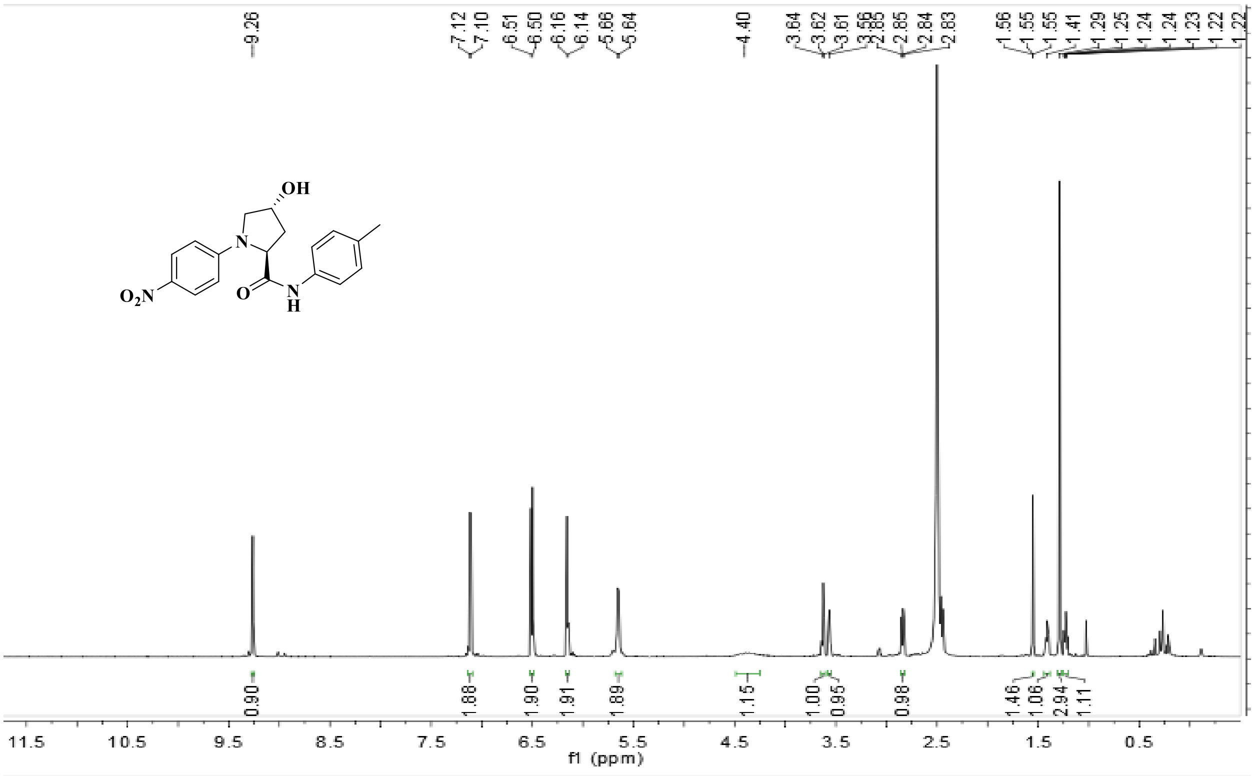

^{13}C-NMR spectrum of *trans*-4-Hydroxy-*N*-(4'-nitrophenyl)-*N'*-(4''-tolyl)-L-prolinamide (4m)

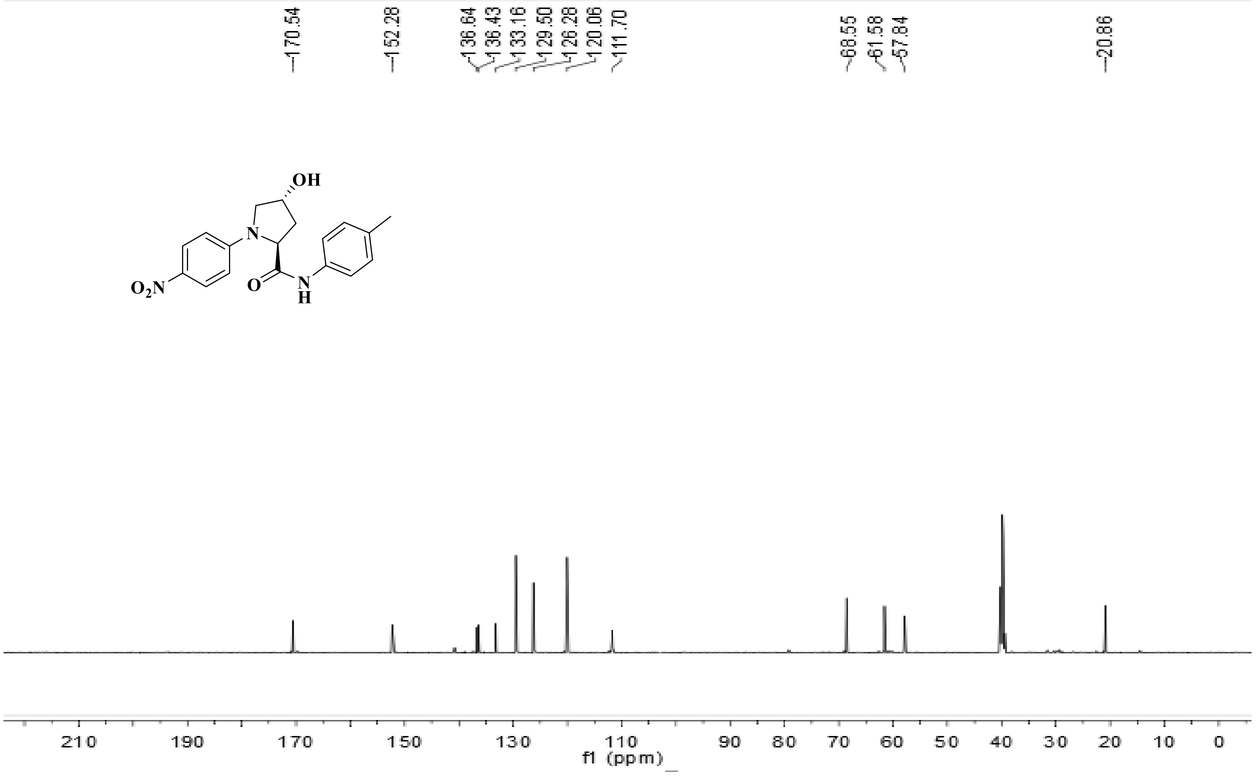

^{1}H-NMR spectrum of 2''-Isoindolinyl *N*-(4'-nitrophenyl)-2-pyrrolidinyl ketone (4n)

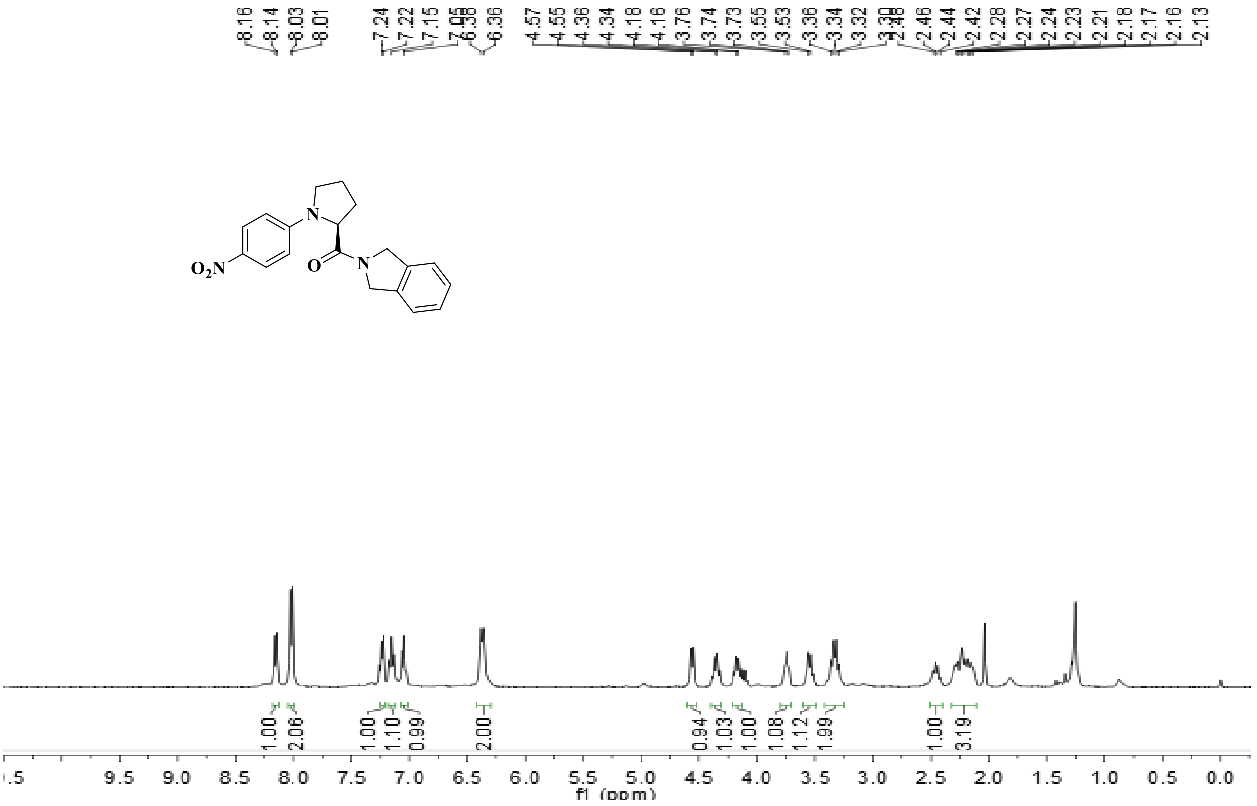

^{13}C-NMR spectrum of 2''-Isoindolinyl *N*-(4'-nitrophenyl)-2-pyrrolidinyl ketone (4n)

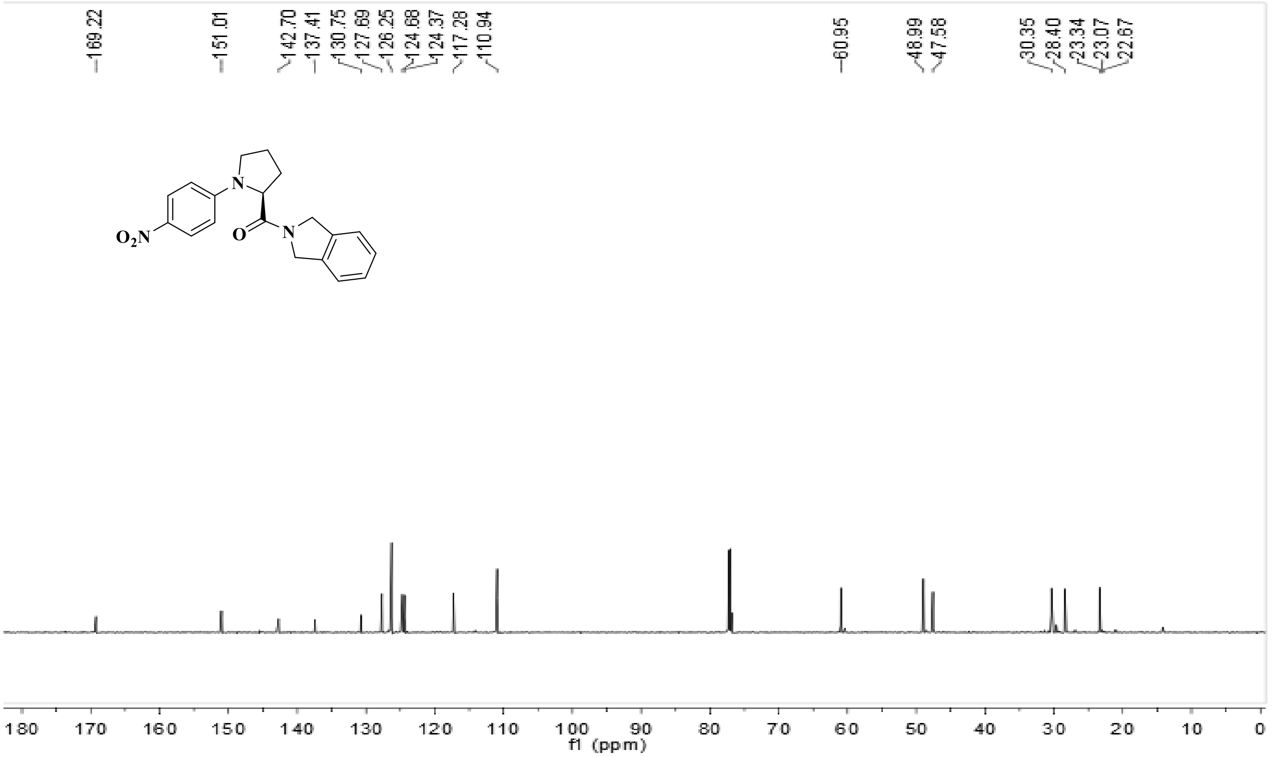

^{1}H-NMR spectrum of *N*-(4'-Nitrophenyl)-*N'*,*N'*-dipropyl-L-prolinamide (4o)

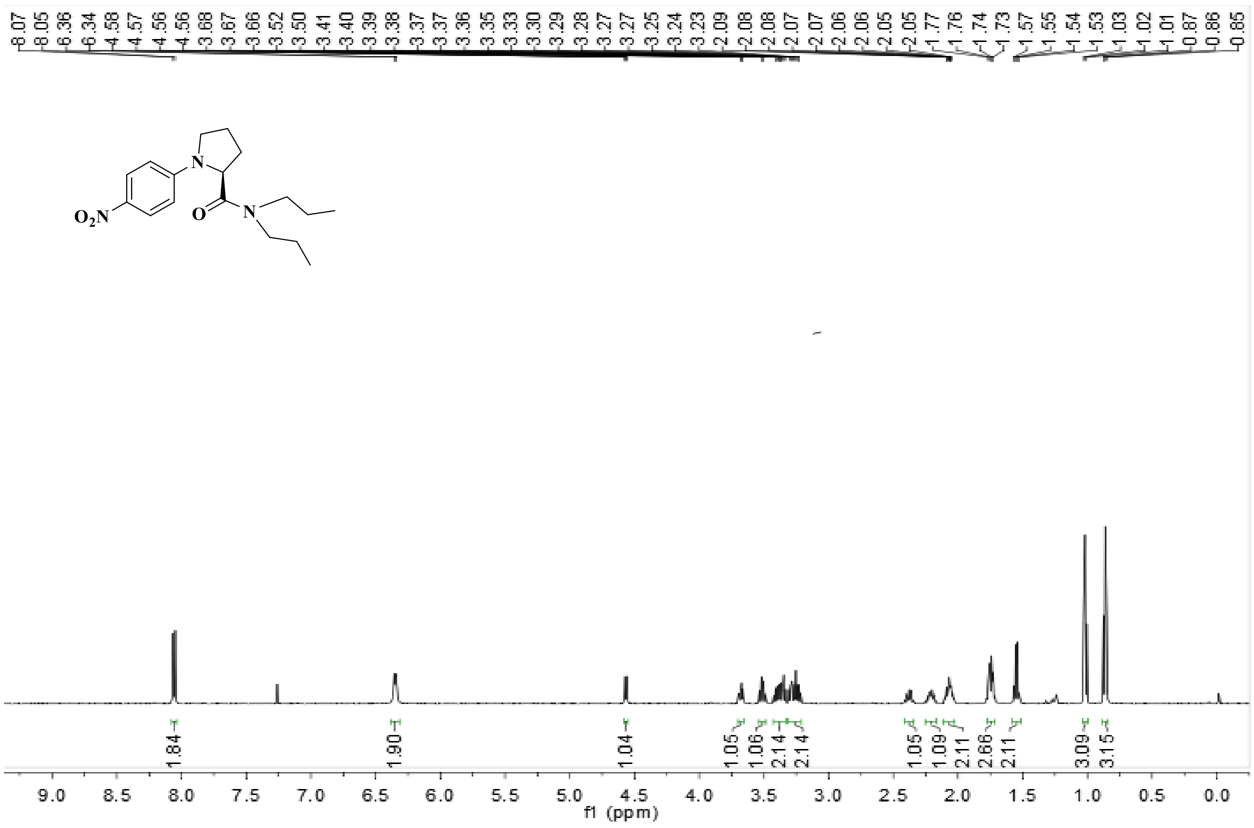

^{13}C-NMR spectrum of *N*-(4'-Nitrophenyl)-*N'*,*N'*-dipropyl-L-prolinamide (4o)

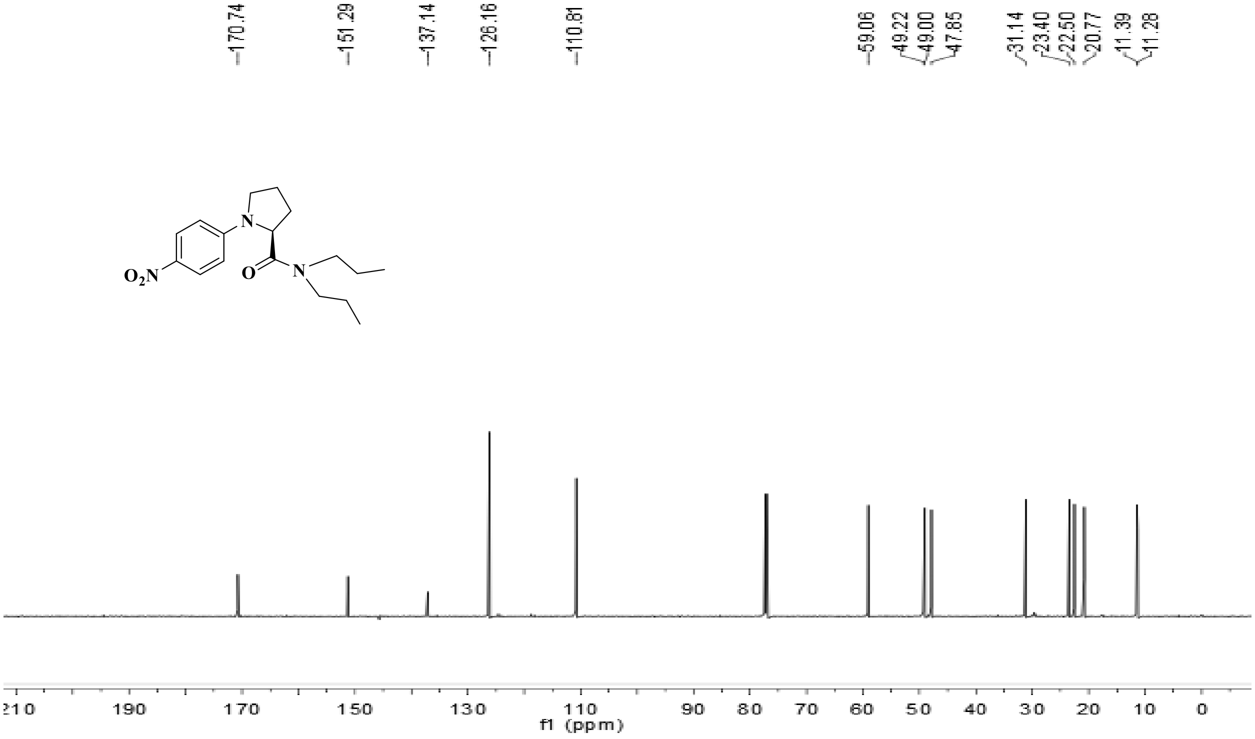

^{1}H-NMR spectrum of *N*-(4'-Nitrophenyl)-*N'*-(2''-pyridinyl)-L-prolinamide (4p)

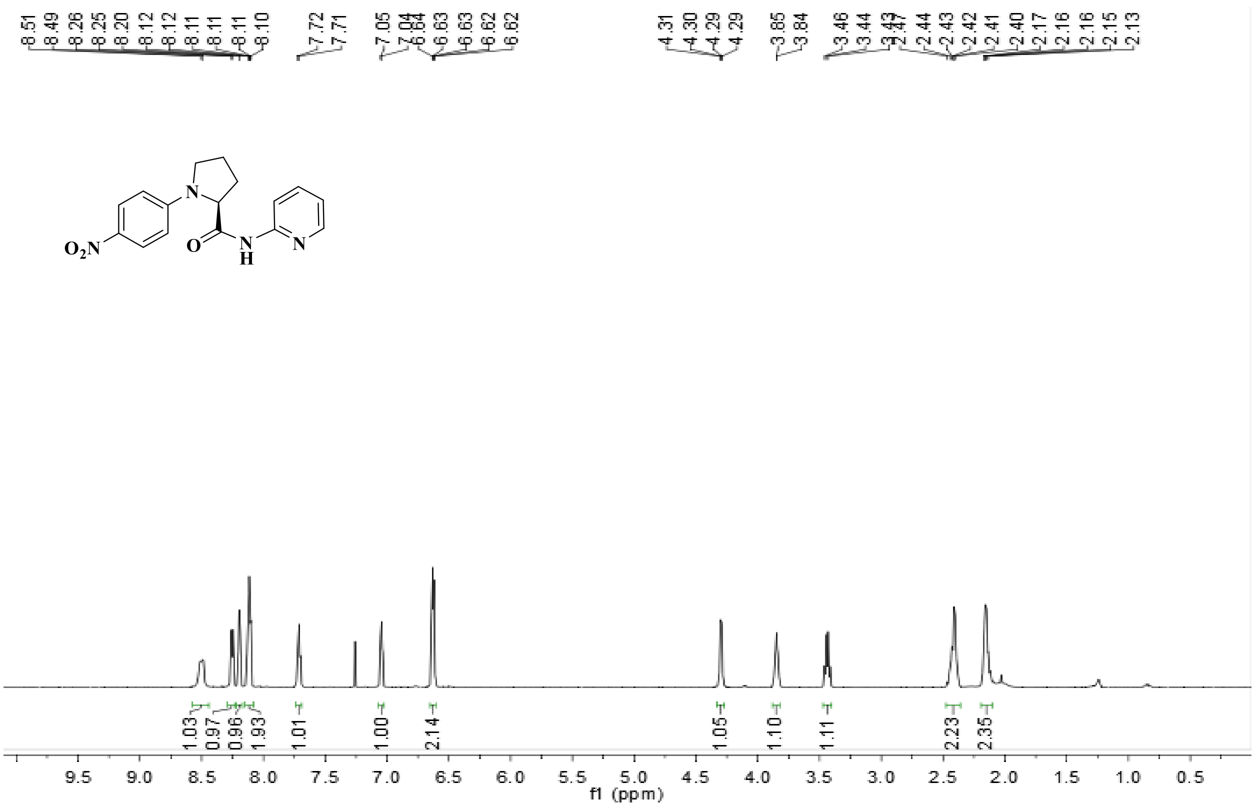

^{13}C-NMR spectrum of *N*-(4'-Nitrophenyl)-*N'*-(2''-pyridinyl)-L-prolinamide (4p)

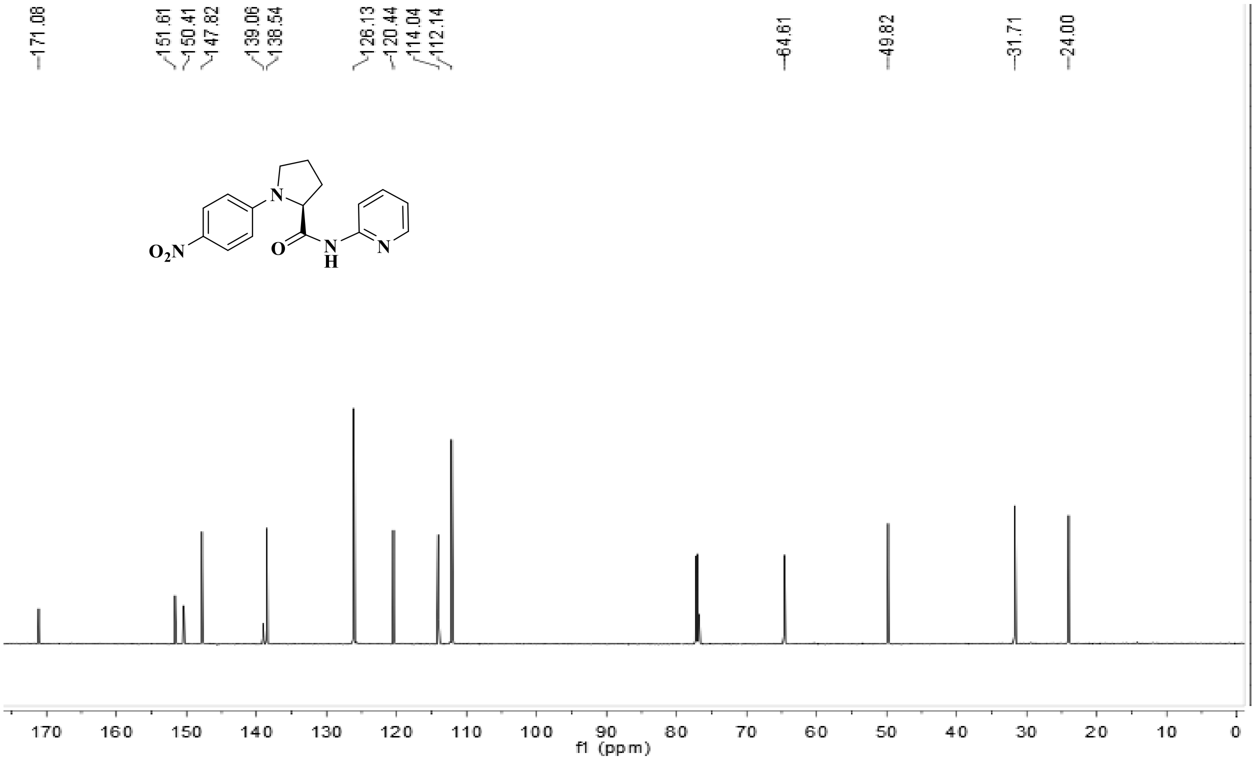

^{1}H-NMR spectrum of *N*'-Benzyl-*N*-(4'-nitrophenyl)-L-prolinamide (4q)

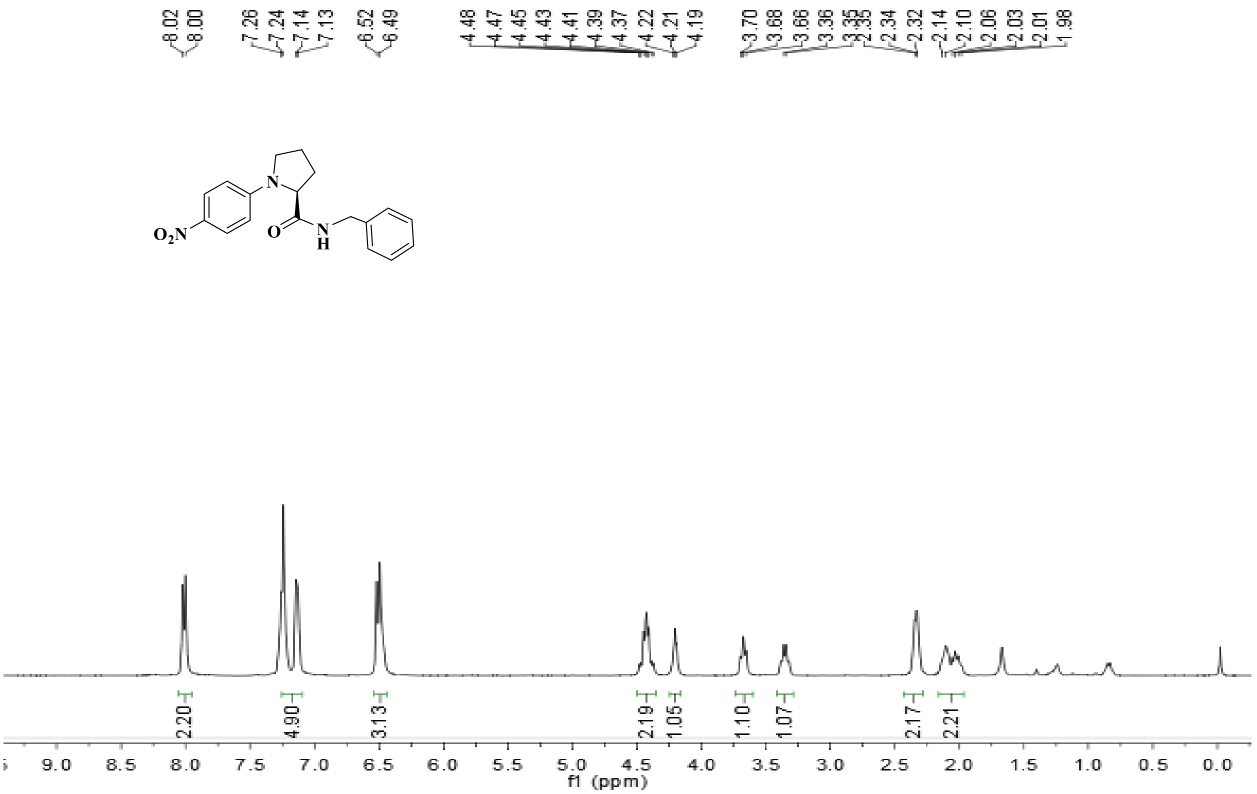

^{13}C-NMR spectrum of *N*'-Benzyl-*N*-(4'-nitrophenyl)-L-prolinamide (4q)

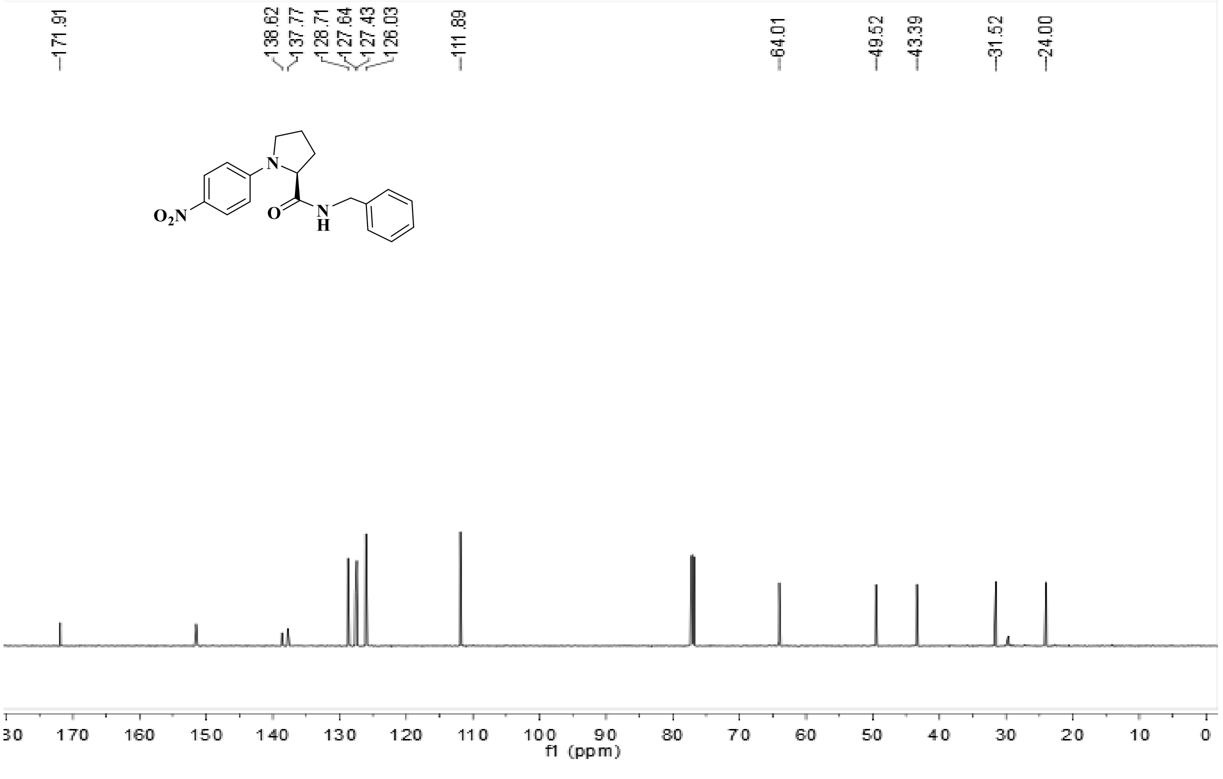

^{1}H-NMR spectrum of *N'*-(2''-Cyanophenyl)-*N*-(4'-nitrophenyl)-L-prolinamide (4r)

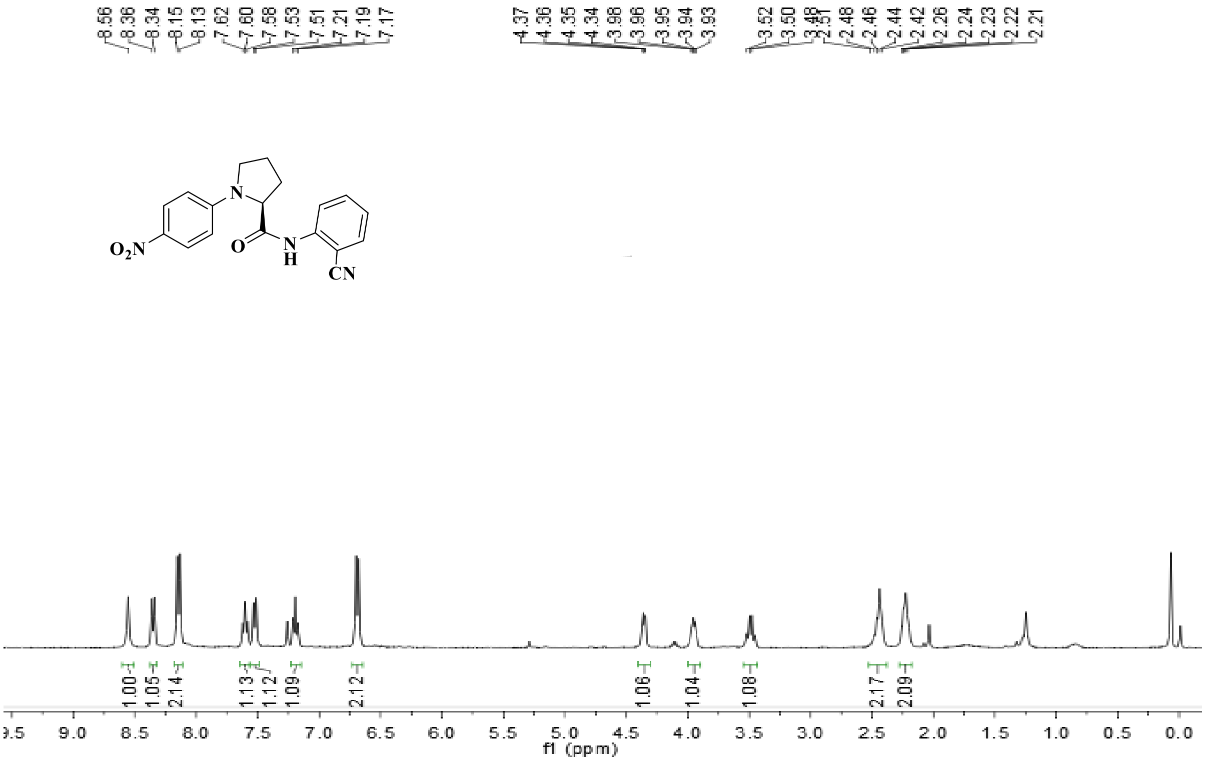

^{13}C-NMR spectrum of *N'*-(2''-Cyanophenyl)-*N*-(4'-nitrophenyl)-L-prolinamide (4r)

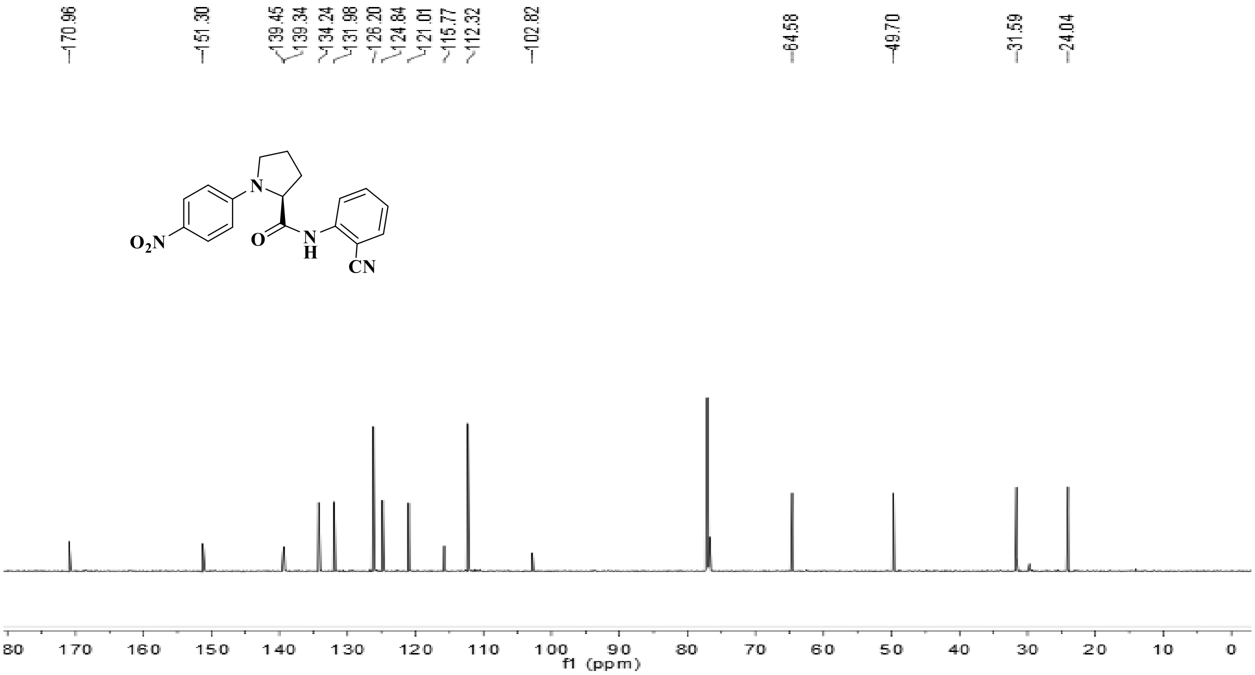

^{1}H-NMR spectrum of *N'*-Methyl-*N*-(4'-nitrophenyl)-*N'*-phenyl-L-prolinamide (4s)

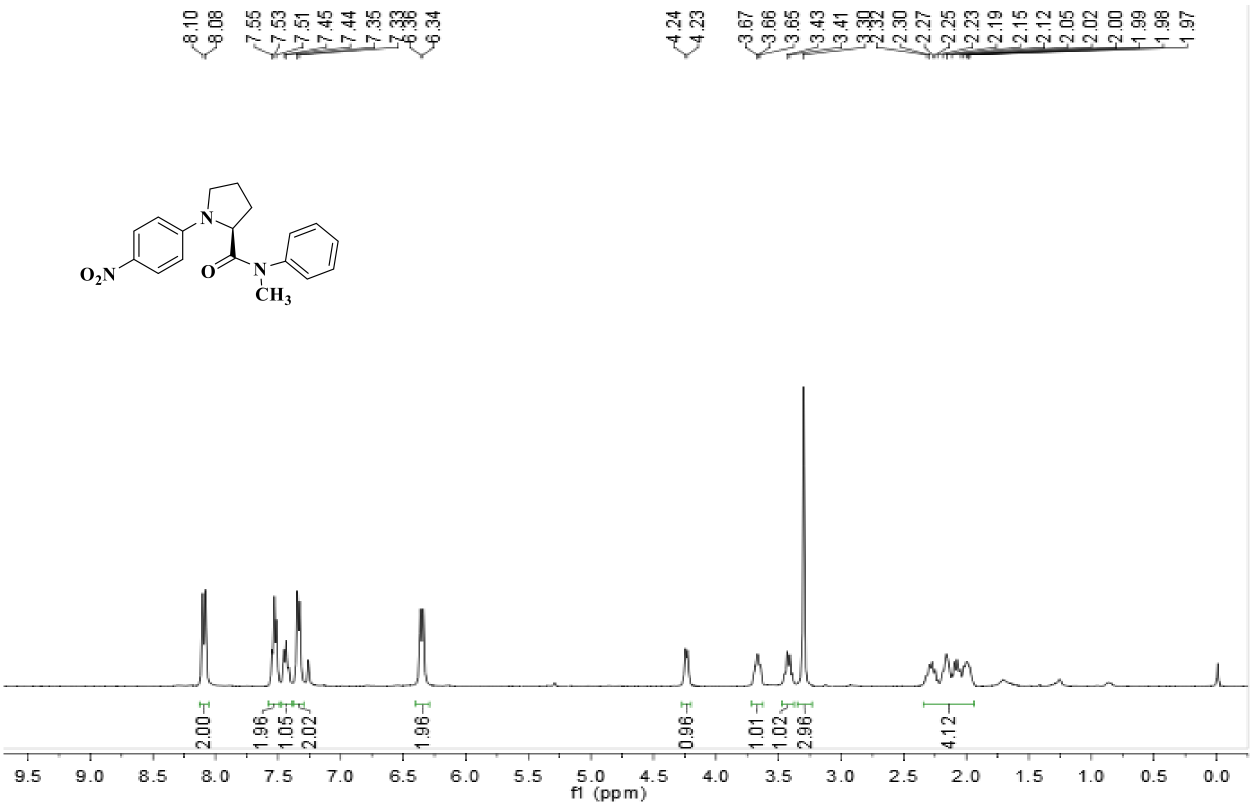

^{13}C-NMR spectrum of *N'*-Methyl-*N*-(4'-nitrophenyl)-*N'*-phenyl-L-prolinamide (4s)

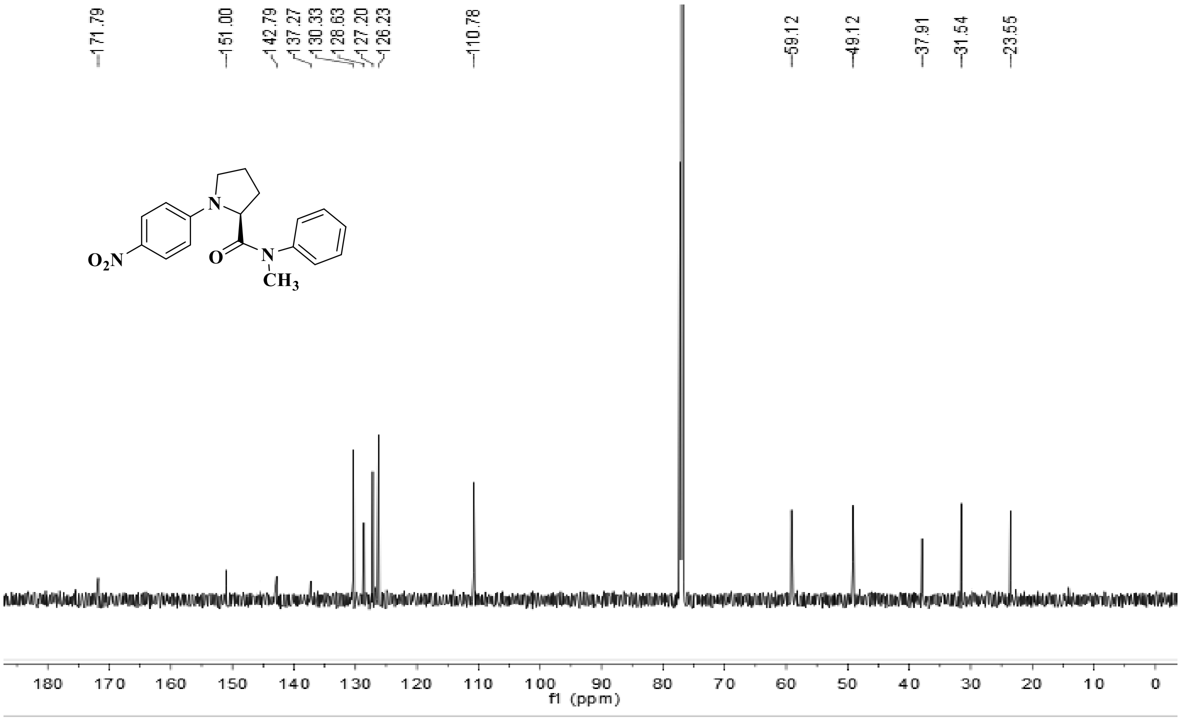

^{1}H-NMR spectrum of *N'*-(2''-(Hydroxymethyl)phenyl)-*N*-(4'-nitrophenyl)-L-prolinamide (4t)

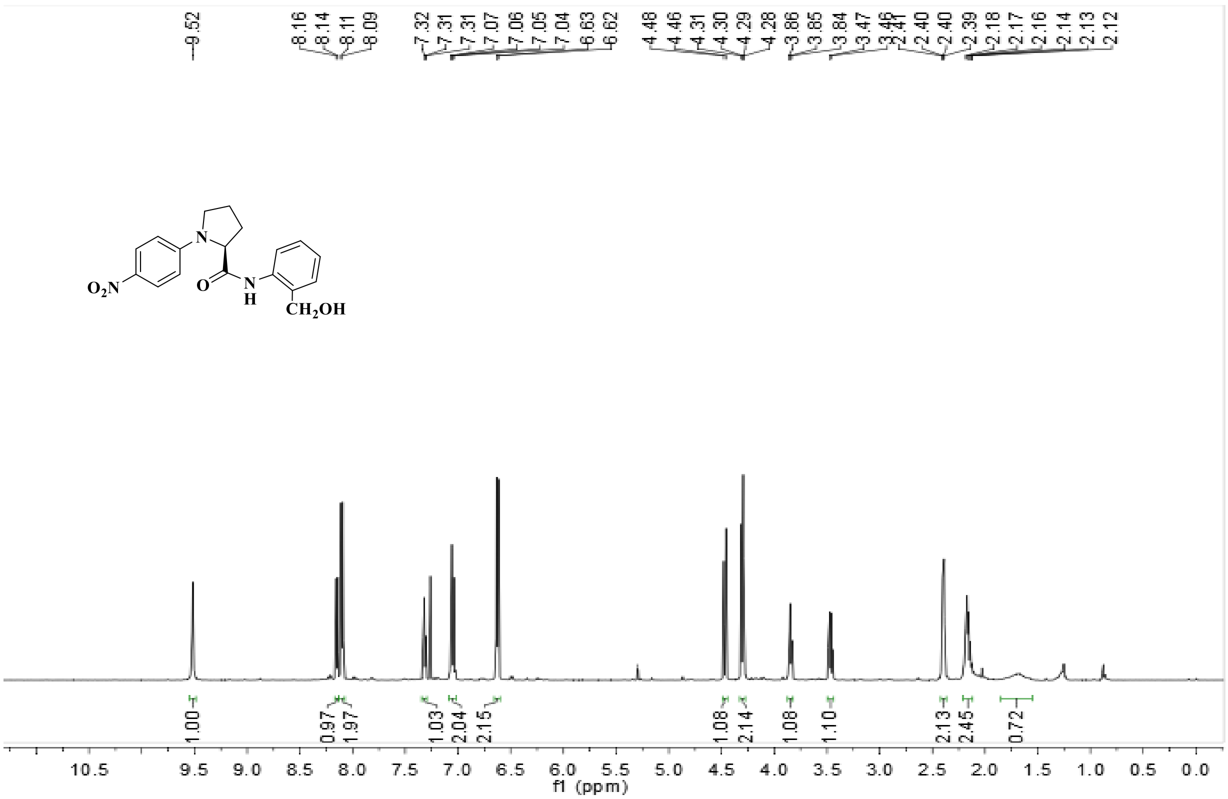

^{13}C-NMR spectrum of *N'*-(2''-(Hydroxymethyl)phenyl)-*N*-(4'-nitrophenyl)-L-prolinamide (4t)

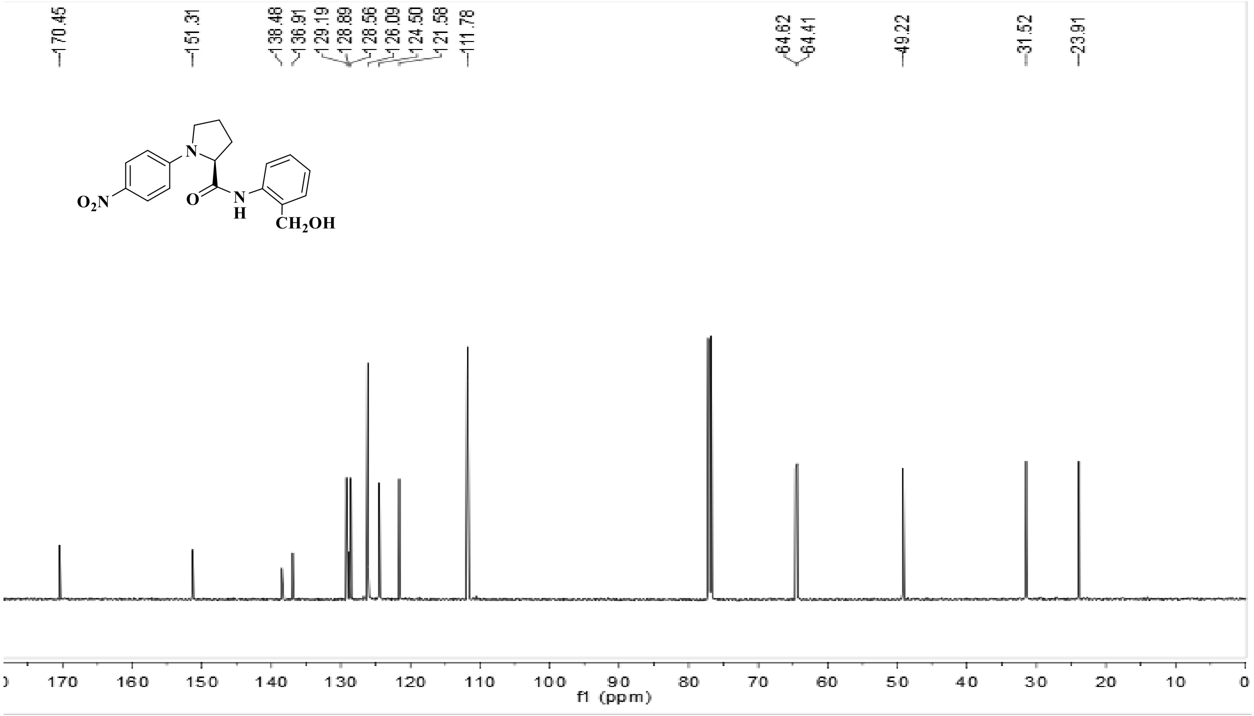

^{1}H-NMR spectrum of *N,N'-bis*(4'-Nitrophenyl)-L-prolinamide (4u)

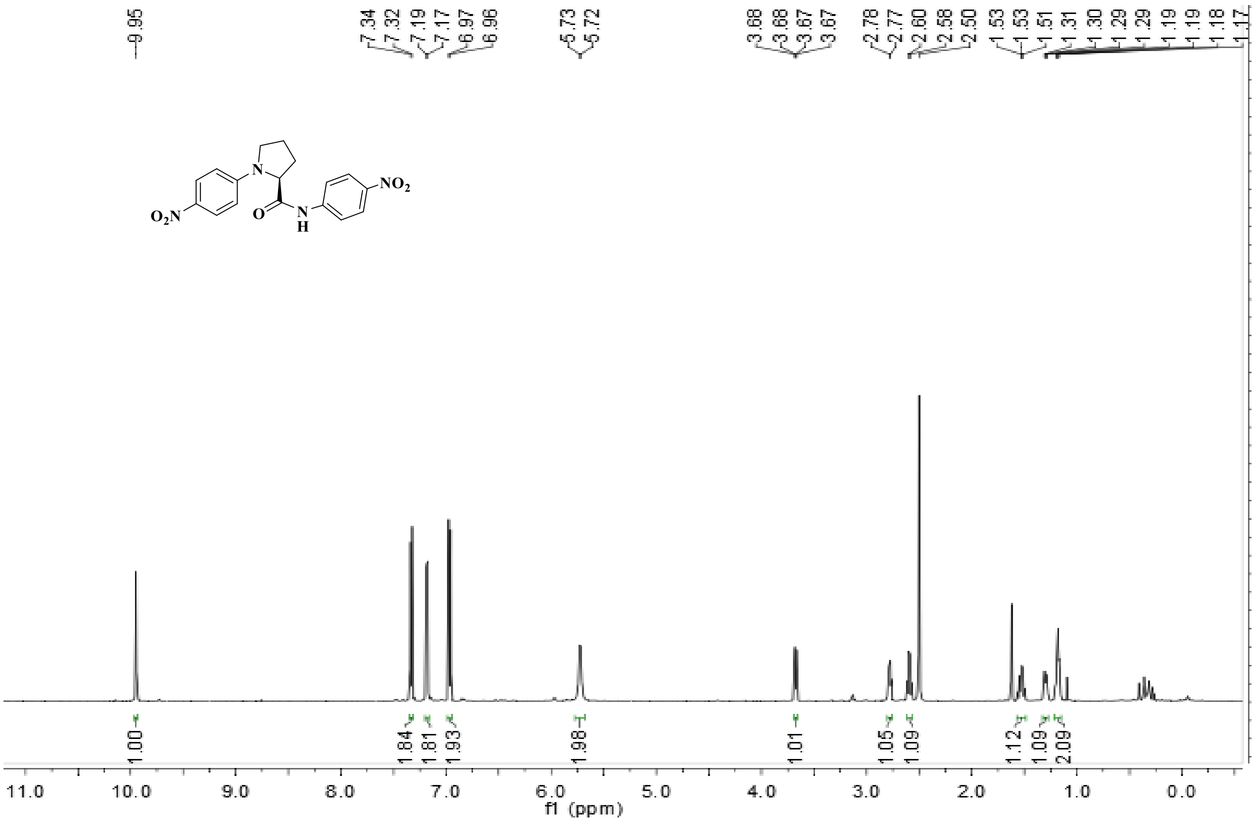

^{13}C-NMR spectrum of *N,N'-bis*(4'-Nitrophenyl)-L-prolinamide (4u)

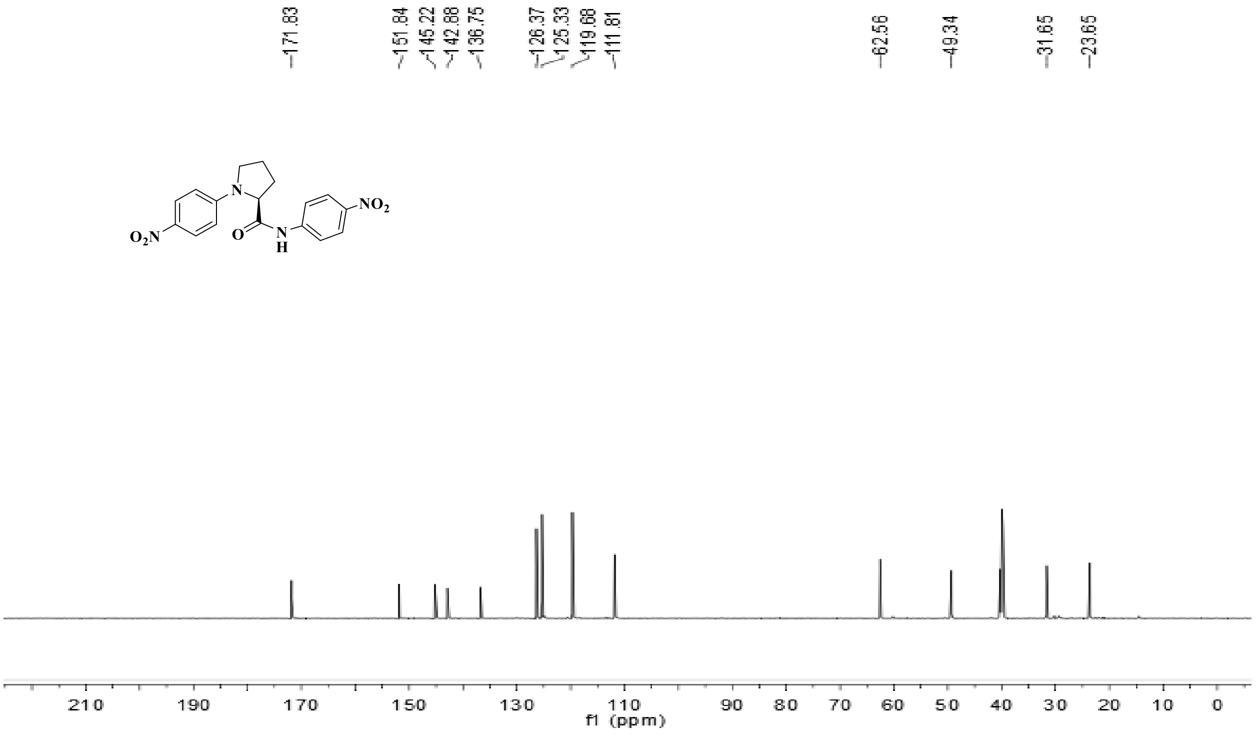

^{1}H-NMR spectrum of *N'*-Isopropyl-*N*-(4'-nitrophenyl)-L-prolinamide (4v)

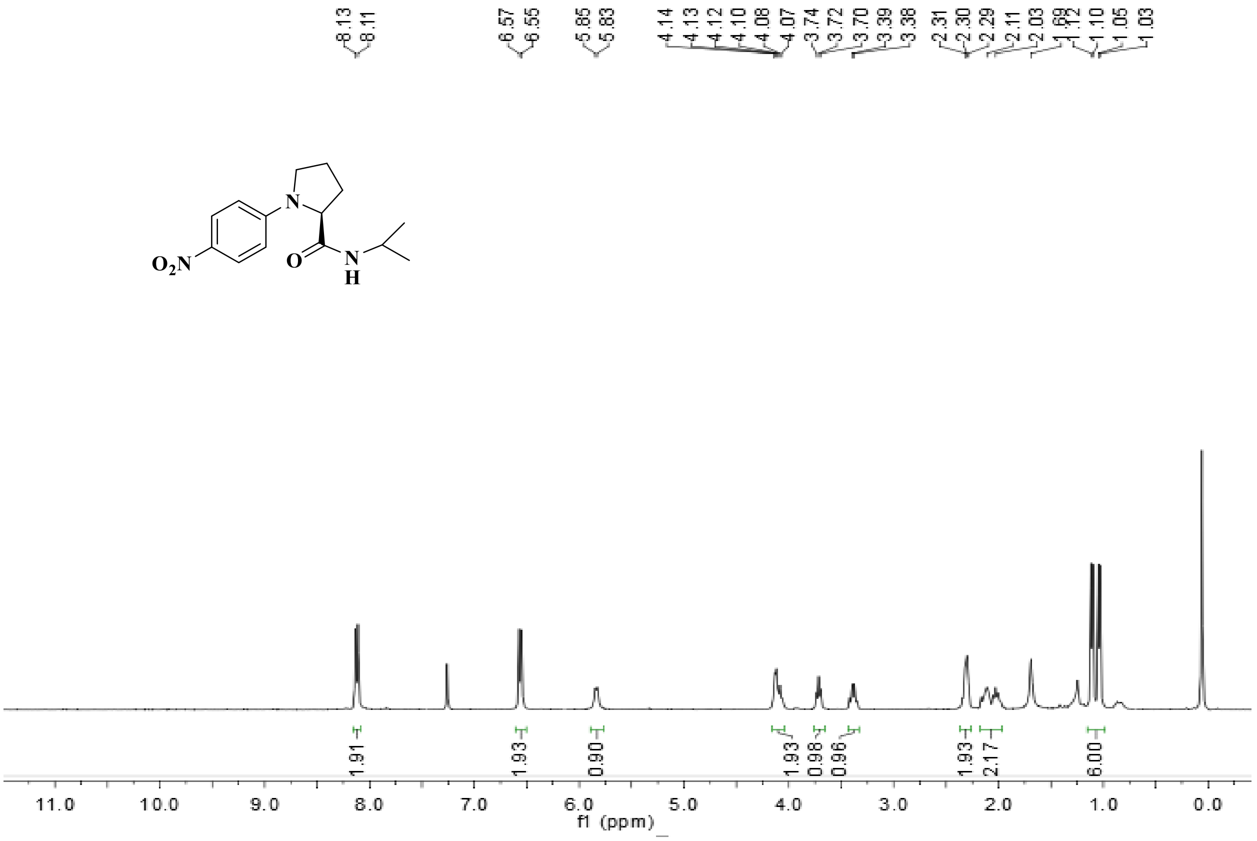

^{13}C-NMR spectrum of *N'*-Isopropyl-*N*-(4'-nitrophenyl)-L-prolinamide (4v)

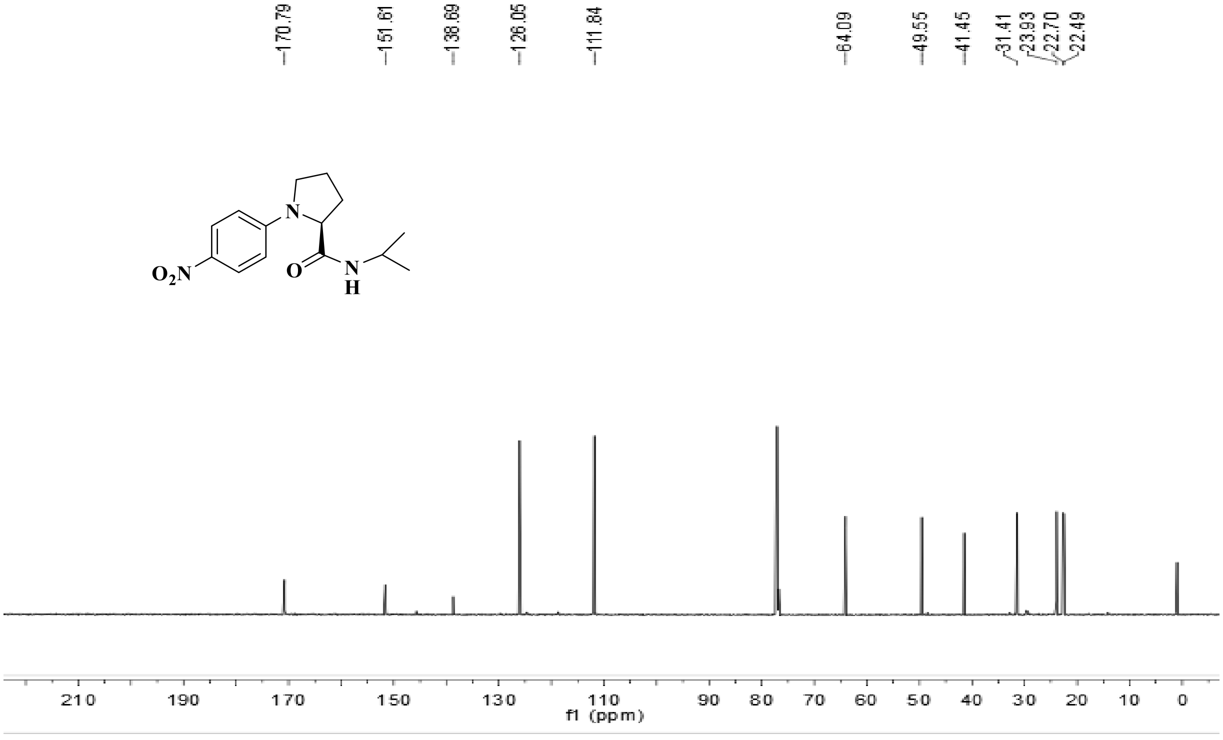

^{1}H-NMR spectrum of *N'*,*N'*-Dicyclohexyl-*N*-(4'-nitrophenyl)-L-prolinamide (4w)

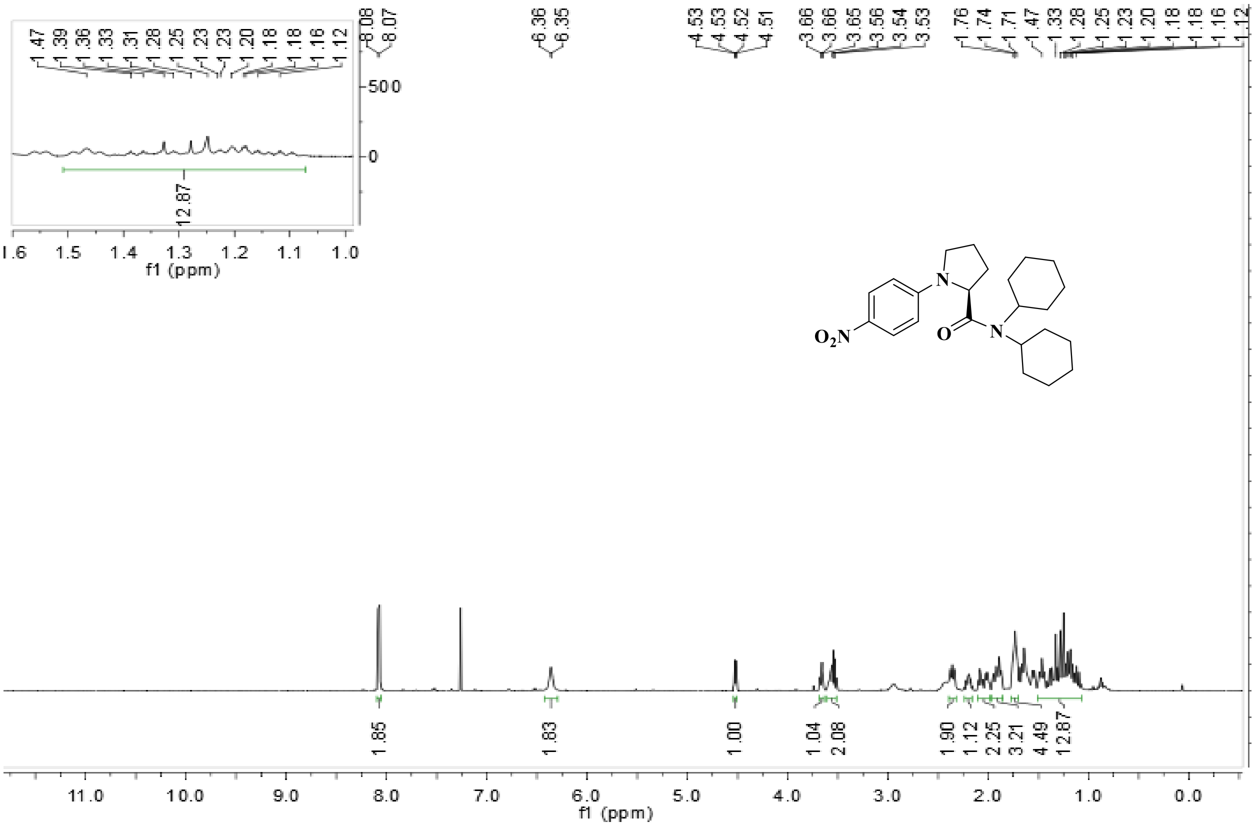

^{13}C-NMR spectrum of *N'*,*N'*-Dicyclohexyl-*N*-(4'-nitrophenyl)-L-prolinamide (4w)

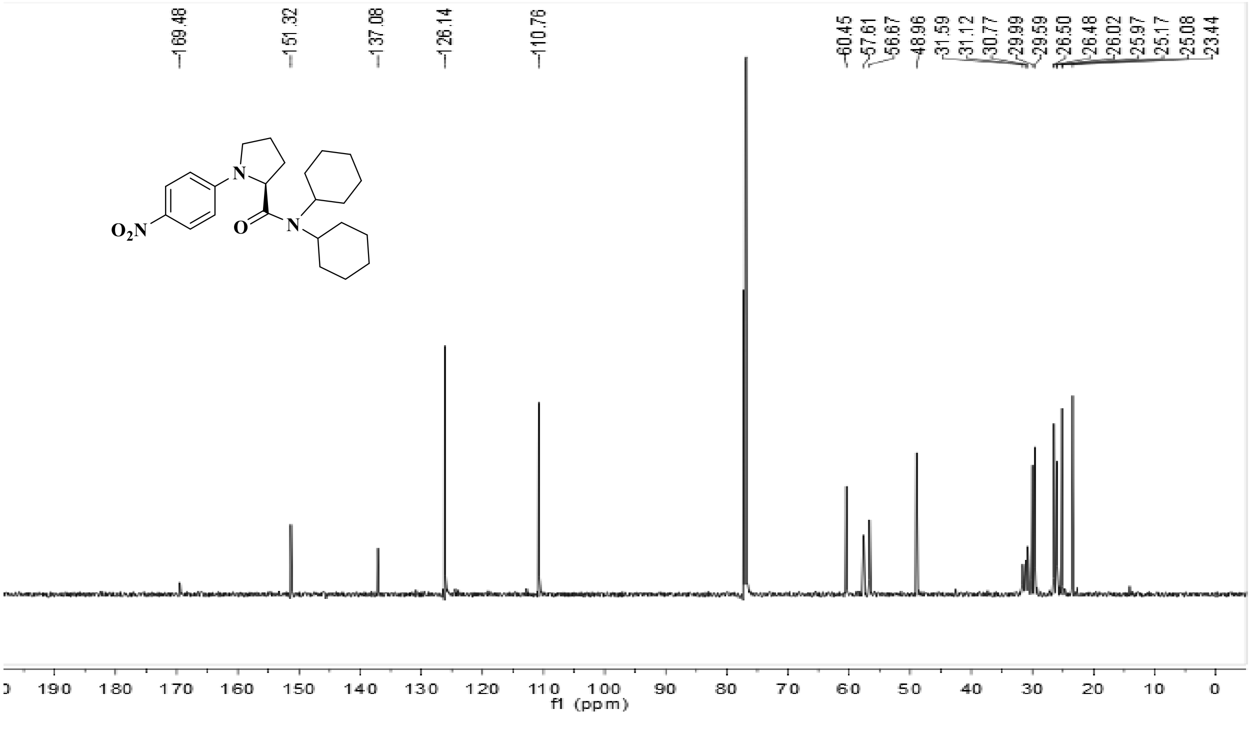

Supplement: Supporting Information [file rsos200906supp1.pdf]
